# The AGMK1-9T7 cell model of neoplasia: Evolution of DNA copy-number aberrations and miRNA expression during transition from normal to metastatic cancer cells

**DOI:** 10.1371/journal.pone.0275394

**Published:** 2022-10-24

**Authors:** Andrew M. Lewis, Rachael Thomas, Matthew Breen, Keith Peden, Belete Teferedegne, Gideon Foseh, Alison Motsinger-Reif, Daniel Rotroff, Gladys Lewis

**Affiliations:** 1 Laboratory of DNA Viruses, Division of Viral Products, Center for Biologics Evaluation and Research, Food and Drug Administration, Silver Spring, MD, United States of America; 2 Department of Molecular Biomedical Sciences, College of Veterinary Medicine, and Center for Comparative Medicine and Translational Research, Raleigh, NC, United States of America; 3 Bioinformatics Research Center, Department of Statistics, North Carolina State University, Raleigh NC, United States of America; 4 TCL and M Associates, Leesburg, VA, United States of America; University of Tennessee Health Science Center, UNITED STATES

## Abstract

To study neoplasia in tissue culture, cell lines representing the evolution of normal cells to tumor cells are needed. To produce such cells, we developed the AGMK1-9T7 cell line, established cell banks at 10-passage intervals, and characterized their biological properties. Here we examine the evolution of chromosomal DNA copy-number aberrations and miRNA expression in this cell line from passage 1 to the acquisition of a tumorigenic phenotype at passage 40. We demonstrated the use of a human microarray platform for DNA copy-number profiling of AGMK1-9T7 cells using knowledge of synteny to ‘recode’ data from human chromosome coordinates to those of the African green monkey. This approach revealed the accumulation of DNA copy-number gains and losses in AGMK1-9T7 cells from passage 3 to passage 40, which spans the period in which neoplastic transformation occurred. These alterations occurred in the sequences of genes regulating DNA copy-number imbalance of several genes that regulate endothelial cell angiogenesis, survival, migration, and proliferation. Regarding miRNA expression, 195 miRNAs were up- or down-regulated at passage 1 at levels that appear to be biologically relevant (i.e., log2 fold change >2.0 (q<0.05)). At passage 10, the number of up/down-regulated miRNAs fell to 63; this number increased to 93 at passage 40. Principal-component analysis grouped these miRNAs into 3 clusters; miRNAs in sub-clusters of these groups could be correlated with initiation, promotion, and progression, stages that have been described for neoplastic development. Thirty-four of the AGMK1-9T7 miRNAs have been associated with these stages in human cancer. Based on these data, we propose that the evolution of AGMK1-9T7 cells represents a detailed model of neoplasia in vitro.

## Introduction

The investigation of spontaneous neoplastic development/transformation (SPNDT) in tissue culture represents the study of a fundamental process in biology. Current studies of neoplasia generally focus on the end-stage of this process, namely cancer, which represents the most diverse and complex aspect of neoplasia. Although the tissue-culture model, which was developed in the early 1940s [1, 2], is no longer a primary focus of the study of neoplasia, it represents one of the few systems available for the systematic evaluation of neoplastic development over time in the same population of cells. The critical aspect of the tissue-culture model is the ability to cryopreserve cell samples for future studies at any point during serial passage [3, 4]. This allows the examination of the neoplastic process at any stage of its evolution from normal (non-immortal, non-tumorigenic) cells to immortal cells that evolve to express a tumorigenic phenotype [5].

The VERO line of African green monkey kidney (AGMK) cells was established in the early 1960s by Yasumura and Kawakita to study simian virus 40 (SV40) [6]. Subsequent studies found that VERO cells supported the replication of a wide variety of viruses and that these cells expressed an immortalized, non-tumorigenic phenotype even after more than 160 passages (p) in culture [7, 8]. These characteristics were among those that made the VERO cell line useful for viral diagnostics and for viral-vaccine production [9–11]. Our studies have shown that the World Health Organization (WHO) 10–87 VERO cell line can be used for the evaluation of the biology of SPNDT in non-human primate cells [12, 13]. During these studies using 10–87 VERO cells, we detected the expression of four miRNAs as potential biomarkers associated with the acquisition of a tumorigenic phenotype [13].

As passages of VERO cells below p123 were not available, we generated the AGMK1-9T7 cell line and established working cell banks at 10-passage intervals. Our approach was similar to the one we described for the development of canine kidney cell lines [5]. These canine kidney cell lines provided an opportunity to analyze the patterns of chromosomal DNA copy-number aberrations (CNAs) across SPNDT. Details of the initial work describing the AGMK1-9T7 cell line and expression of potential biomarkers of the VERO/AGMK cell tumorigenic phenotype have been reported [14]. The expression of six potential biomarker miRNAs expressed by tumorigenic 10–87 VERO cells and confirmed by RT-qPCR were reported by Teferedegne et al. [15]; four additional potential biomarkers (detected by RT-qPCR) for a total of ten were expressed by AGMK1-9T7 cells at p40 [14]. The genes for these ten potential biomarkers were located on the monkey chromosome equivalent to human chromosome 14q32/31 (MIR Gene Cards–The Human Gene Database), which might indicate the association of these new miRNAs with the tumor-forming ability of 10–87 VERO and AGMK1-9T7 cells [16, 17].

In this report, we focus on the evolution of AGMK1-9T7 cells from normal cells to tumorigenic/metastatic cells, describe the progressive accumulation of chromosomal DNA copy-number aberrations (CNAs), and identify profiles of miRNA expression across the spectrum of SPNDT in tissue culture. The miRNA profiles in the AGMK1-9T7 cell lines evolved dramatically. Large numbers of miRNAs up- or down-regulated at >2-fold changes were expressed at p1/p2, their numbers were sharply reduced at p10, but subsequently increased from p10 to p40 during the evolution across the spectrum of neoplasia. miRNAs have been shown to play major roles in regulating gene expression, and the expression of specific miRNAs has been associated with tumor development in human and non-human species [18]. While there is discussion of the role miRNAs play in neoplasia, the association of specific miRNAs with the capacity of AGMK1-9T7 cells at p40 to form tumors suggests that expression of these miRNAs may be involved in neoplastic transformation in tissue culture.

Based on the DNA CNAs and expression of miRNAs in cells from six passages of the AGMK1-9T7 cell line (p1, p2, p10, p20, p30, p40), we propose that the evolution of the AGMK1-9T7 cells in culture represents a model of SPNDT *in vitro* and that the different passages of the AGMK1-9T7 cell line represent the first detailed *in vitro* model of neoplasia from normal cells to tumor/metastatic cells.

## Materials and methods

### Cell cultures

As were the AGMK cells used to establish the VERO cell line [19], the AGMK cells used in these studies were obtained from the kidney of a female African green monkey (AGMK1). The AGMK cells were obtained as a suspension containing 20 x 10^6^ cells/mL from Diagnostic Hybrids (Athens, OH; lot No. C-460921). To generate baseline data on miRNA expression, new lots of AGMK cells containing the same concentration of cells from both a female monkey (AGMK2) (lot No. 461005) and a male monkey (AGMK3) (lot No. C461026) were obtained from Diagnostic Hybrids. Many of the details on the derivation and characterization of the AGMK1-9T7 cells have been described [14]. AGMK1 cells were established in culture and passaged in Dulbecco’s Modified Eagle’s Medium (Mediatech Inc., Manassas, VA) with 10% fetal bovine serum (FBS) (Hyclone; Thermo Scientific, Logan UT) and 2 mM glutamine (DMEM-10) in T150 flasks (Corning, Corning, NY), and a cell bank was generated at p3. To initiate the establishment of the AGMK1-9T7 cell line, cells from the p3 cell bank were thawed, plated at 9 x 10^5^ cells/flask (1.2 x 10^4^ cells/cm^2^) in six T75 flasks and passaged at low density (estimated to be 95% confluent) at 7-day intervals. Using this procedure, these cells were carried from p3 to p40 with cell banks established every 10 passages. No cytopathology was observed by light microscopy during the passage of these cells. All the cells used in this study were documented to be of simian origin by karyology and were free of 31 contaminating agents including 5 strains of mycoplasma (IDEXX BioAnalytics, Columbia, MO).

### DNA copy-number analysis

Genomic DNA was isolated from cell pellets obtained from cultured AGMK1-9T7 cells at passages 3, 11, 22, 31 and 41 (Qiagen DNeasy Blood and Tissue Kit; Qiagen, Valencia, CA). Genome-wide oaCGH analysis was performed as described previously [20–24] using human microarray platforms due to the absence of AGM-specific resources. To validate this approach, a self-self hybridization of AGMK1-9T7 cells at passage 3 was carried out using a microarray comprising ~410,000 unique, repeat-masked ~60-mer oligonucleotide probes distributed at approximately 6-kb intervals throughout the Genome Reference Consortium Human Build 38 sequence assembly (hg38, Design ID 021850, Agilent Technologies, Santa Clara, CA). A second microarray (Design ID 022060, comprising ~170,000 unique probes distributed at approximately 15-kb intervals throughout hg38) was then used for independent, pairwise hybridizations of AGMK1-9T7 cells at passage 3 (labeled with Cyanine-5-dUTP) with each of the 4 later passages (Cyanine-3-dUTP). Data normalization and filtering were performed using Feature Extraction version 10.10 (Agilent Technologies), and genomic imbalances were identified using the FASST2 segmentation algorithm of Nexus Copy Number version 10 (Biodiscovery, El Segundo, CA). Discrete regions of DNA CNAs in later passage cells (p11, p22, p31 or p41), relative to the baseline passage 3 cells, were defined as 3 or more consecutive probes exhibiting log_2_ values ≥ 0.2 (gain) or ≤ -0.2 (loss). High-amplitude gains and losses were defined using default log_2_ test: reference values of >1.14 and <-1.1, respectively. Data output in this format was used to evaluate the gene content of regions of recurrent CNA, using the human ref Seq track associated with hg38 (https://genome.ucsc.edu), and to enable direct comparison with data from human studies. The LiftOver Batch Coordinate Conversion tool (https://genome-store.ucsc.edu/) was used to convert the human genome coordinate for each arrayed probe into its equivalent within the reference genome of the African green monkey [25], which allowed data to be interpreted in context with the AGM karyotype. Each CNA is reported according to its AGM chromosomal location, as defined by the start and end position in the chlSab2 genome assembly (*Chlorocebus sabaeus* is the scientific name for the African green monkey: e.g., CAE 1: 1.5–2.0Mb). For key regions of interest, the broadly equivalent interval within the human hg38 genome assembly is also defined (e.g., HSA 1:1.5–2.0Mb), based on cross-species mapping of large blocks of evolutionarily conserved synteny [26] using the LiftOver tool (genome.ucsc.edu/cgi-bin/hgLiftOver). (Abbreviations: CAE is *Chlorocebus sabaeus*; HSA is *Homo sapiens)*

### Basic information regarding RNA preparation and miRNA assays

Defining the profiles of miRNA expression as cells evolve from normal to tumorigenic should identify a basic component of the neoplastic process in tissue culture.

There are at least three types of cells present in the kidney. First are the suspension cells created by the enzymatic digestion of the kidney tissues; these suspensions would also contain cells from the blood that were present in the kidney when it was digested. Second are the cells that survive when the suspension cells are seeded on plastic tissue-culture flasks; these are cells defined as passage (p) 0. Third are the cells that become the first passage in culture; these are cells at p1. Evidence presented in this report indicates that the process of cellular evolution to survive in culture represents the beginning of the process of neoplastic development as the cells evolve to express a tumorigenic phenotype, that is, have the capacity to form tumors in immunodeficient rodents. A fundamental component of, and scientific questions about, the process of neoplastic development in vitro is when it begins. The decision to use cells in suspension as the baseline for this report was due to the need to characterize the full spectrum of changes as these cells adapted to the tissue-culture environment and became neoplastic. We recognize that some of the changes that occurred from suspension to p0 to p1 may be cell-type specific or be due to an adaptive response unrelated to neoplasia. However, given that the exact time the cells growing in tissue culture initiate the process of neoplasia is unknown, we wanted to be inclusive and to capture all changes.

Global miRNA analysis was performed on AGMK1-9T7 cells at p1, p2, p10, p20, p30, and p40. RNA was extracted using the Qiagen mRNeasy kit (Qiagen, Rockville, MD) following the supplier’s instructions. Microarray-based profiling was performed using a service provider (LC Sciences, Houston, TX) as described previously [13]. Because the genome sequence of the African green monkey was not available when the assays were done, and there were no commercially available African green monkey miRNA chips, a primate miRNA chip that contained fewer than 2,300 human [27] and 500 non-human [28] primate miRNA transcripts in the Sanger miRBase Release 15.0 was used (http://mirbase.org).

### miRNA data processing and statistical analysis

All analyses were performed using the open-source, statistical software (R Core Team, 2020: A language and environment for statistical computing; this was from the R Foundation for statistical computing, Vienna Austria. URL: https//www.R-project.org). First, technical replicate samples were combined by taking the mean. Next, the fold change of each miRNA at each passage was calculated using the mean of samples in suspension from three different monkeys: AGMK1 (female), AGMK2 (female), and AGMK3 (male). For these calculations, miRNA expression in cell suspensions from the kidneys of five African green monkeys (including the 3 monkeys listed above and two additional AGMK cell suspensions that were obtained after these studies began) was also examined by the same technology. Sixty of the highest levels of up- and down-regulated miRNAs detected were expressed in either all 5 cell suspensions or in 4 out of 5 of these cell suspensions (See S4 Data). Fold-change values were subsequently log_2_ transformed, and individual miRNAs were tested for association with passage using linear-regression models. Because data for p1 and p2 were collected separately from those from cells at later passages, association analyses were conducted separately for these batches to prevent confounding. *P* values were corrected for multiple comparisons using a false-discovery rate (FDR) approach [29]. miRNAs with 2-fold increases or decreases expressed as [| log_2_FC | >2 and q < .05] were considered to be statistically significant. An additional criterion was applied to identify statistically significant miRNAs that were also more likely to be biologically significant. This more stringent requirement to identify miRNAs expressed at biologically relevant levels of expression (BRLE) were those with a q < .05 and either up- and down-regulated [30] with a |log_2_ FC|>2 and are referred to as miRNAs with BRLE. A secondary analysis to identify specific passages with significant changes in miRNA expression was performed. miRNAs that met our criteria for significance based on linear regression were subsequently tested at each passage for miRNA changes with the preceding passage using a Student’s t-test (See S1 Data). *P* values were subsequently corrected for multiple comparisons using the same FDR approach described above. Unsupervised hierarchical clustering of miRNA expression with BRLE was performed using Euclidian distance and average linkage. These data represent the fold-change level of expression of these miRNAs compared with baseline suspension-cell data.

Principal-component analysis (PCA) was performed to determine similarity and to assess cell-line passages based on global miRNA profiles (See S3 Data). Missing values were mean imputed, and replicates across the cell-line passages were collapsed by taking the mean so that a single value for each miRNA was available for each passage combination. Subsequently, miRNA values were centered and scaled, and PCA was performed using *prcomp* in the R statistical language. Plots of the Eigenvectors were created using the *ggfortify* and *factoextra* packages [31, 32].

### Comparison with the miRCancer database

miRNAs that were significantly increased or decreased were compared with miRNAs with cancer associations in the miRCancer Database (Oct 2019 release). Statistical significance was determined using a hypergeometric test (See Tables 1–5 in S2 Data).

## Results

### Cell-line development and characterization

We obtained cell suspensions from the kidneys of three African green monkeys AGMK1 [female], AGMK2 [female], AGMK3 [male] for the generation of a new line of AGMK cells to serve as a baseline for the analysis of the miRNA-expression profiles obtained from both the AGMK1-9T7 and VERO cell lines [13, 14]. Cells from the kidney of a female monkey AGMK1 were used to establish the AGMK1-9T7 cell line; cells from the kidney of a female African green monkey (AGM) were also used to establish the VERO cell line [19]. The cells from the AGMK1 suspension formed cellular colonies during the first 5–6 days in culture and grew into a confluent monolayer by 6 weeks (designated as p0). Beginning at week 7, the cells were passaged at weekly intervals when they had reached approximately 95% confluence, and a cell bank was established at p3. Cells from this bank were passaged at weekly intervals as described in Materials and Methods, with cell banks being established every 10 passages. As described in an earlier report [13], the growth of the AGMK1-9T7 cell lines was associated with a reduction of their doubling times (DT) from 96.7 h at p10 to 18.3 h at p40 [14]. The cells did not become tumorigenic until p40. The cells that grew out exhibited a mesenchymal/fibroblastic morphology during the first 30 passages in culture, but between p30 and p40, the cells underwent what appeared to be mesenchymal-to-epithelial transition (MET) and became predominately epithelial in appearance by p40 [14]. The results of tumorigenicity assays in newborn (NB) and adult (AD) nude mice inoculated with p41 and p44 cells were reported earlier [14].

### Evolution of DNA copy-number aberrations (CNA) during SPNDT

**Fig 1:**
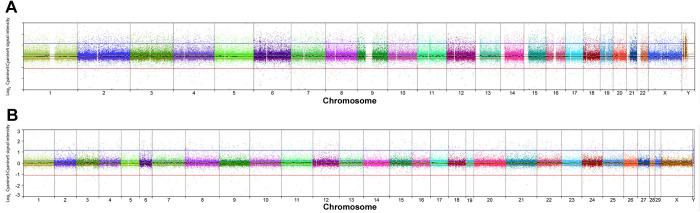
Demonstration that human chromosomes can be used to align DNA copy-number aberrations. **A.** Self-self hybridization of differentially labeled DNA from passage 3 AGMK-9T7 cells onto a human oaCGH microarray. The x-axis indicates the location of each arrayed target sequence along chromosomes 1–22 and the sex chromosomes in the human hg38 reference genome assembly (mean interval 6kb), and the y-axis shows the log_2_ ratio of Cyanine-3:Cyanine-5 fluorescence intensity for each target. Blue and red horizontal lines immediately above and below the midline indicate thresholds for classification of relative DNA copy-number gains and losses, respectively. Datapoints for each chromosome are shown in a different color to aid interpretation. The profile clusters tightly around the midline, consistent with the expected relative balance in fluorescence signal intensity, and no regions of DNA copy-number gain or loss were identified. **B.** The same data plotted against AGM chromosome locations on the x-axis (chromosomes 1–29 and the sex chromosomes), achieved by ‘recoding’ each array human target sequence to define its orthologous position in the chlSab2 reference genome assembly. The increased tightness of the profile around the midline is the result of screening out arrayed human targets for which no robust match could be identified in the AGM genome. The data support the use of human oaCGH platforms for DNA copy number profiling of AGM specimens.

A self-self hybridization was performed using DNA from AGMK1-9T7 cells at p3. This confirmed that human microarrays were capable of generating robust DNA copy-number data from AGM DNA (Fig 1A) by recoding data from the human genome to the AGM genome assembly coordinates (Fig 1B). The results confirmed the validity of this cross-species approach, with profiles of probe hybridization signal quality metrics being broadly consistent with those from our prior data of specimens evaluated on the same array platforms [33, 34].

**Fig 2:**
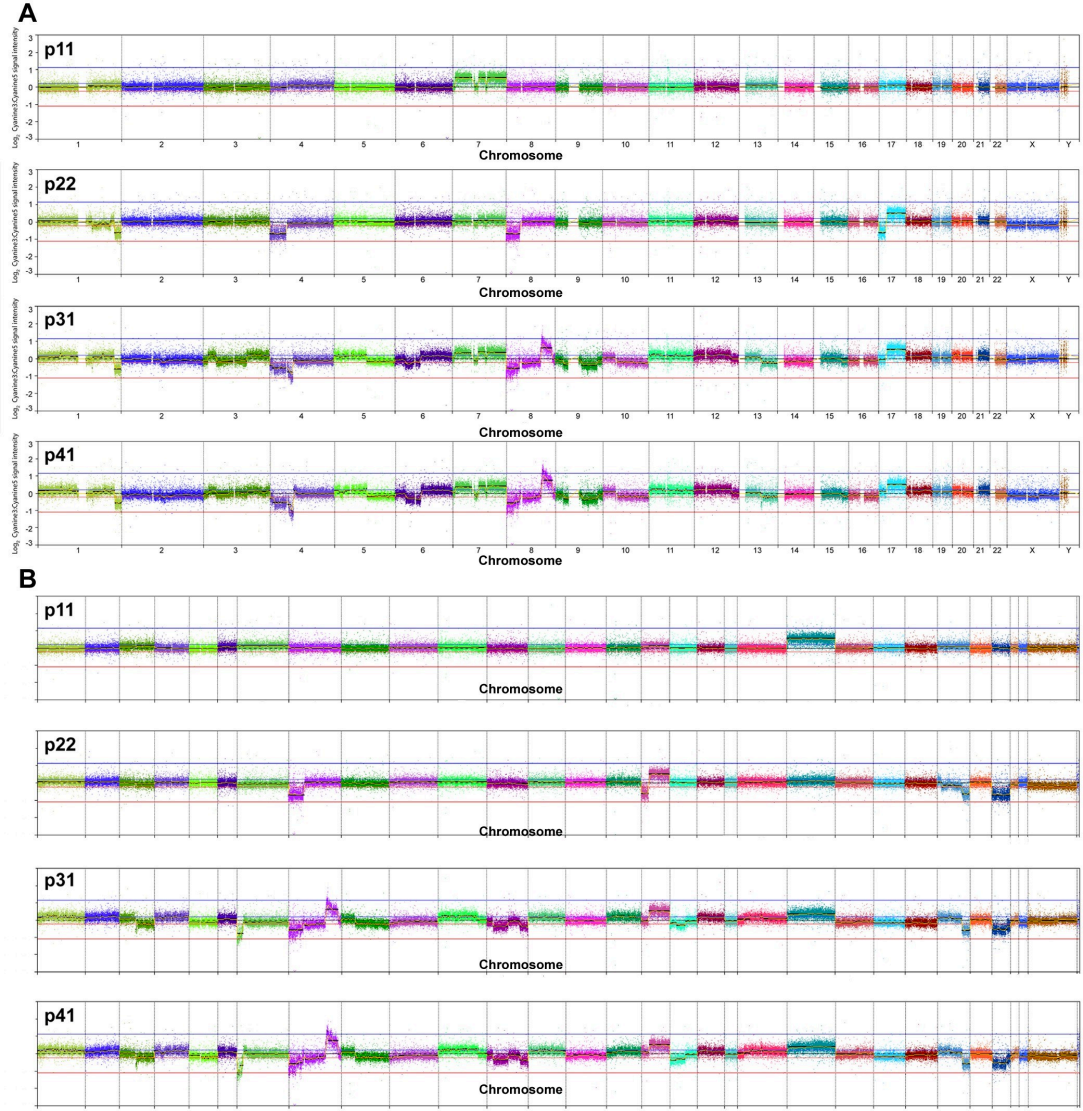
Copy-number profiles of AGMK1-9T7 cells at different passages. **A.** DNA copy-number profiles of four passages of AGMK1-9T7 cells hybridized onto a human oaCGH microarray, relative to a common reference sample (AGMK1-9T7 cells at passage 3). The x-axis indicates the location of each arrayed target sequence in the human hg38 reference genome assembly (mean interval 15 kb), and the y-axis shows the log_2_ ratio of Cyanine-3:Cyanine-5 fluorescence intensity for each target. These profiles show the progressive accumulation of DNA copy-number alterations during continued passage of AGMK1-9T7 cells, comprising a range of genomic gains and losses involving whole chromosome regions, and discrete subchromosomal intervals. **B.** The same data plotted against AGM chromosome locations on the x-axis. The differences in the relative location of DNA copy-number alterations in Fig 2A and 2B reflect the divergent architecture of the human and AGM karyotypes (2n = 22 and 29, respectively).

Subsequent evaluation of later passages of AGMK1-9T7 cells revealed the sequential accumulation of CNAs during propagation in culture, as shown in Fig 2 in both human (Fig 2A) and AGM (Fig 2B) chromosome format. The first CNA to become evident was whole AGM chromosome [*i*.*e*., *Chlorocebus sabaeus* (CAE)] gain of CAE 21, which corresponds to interspersed regions of HSA 7. By p22, this aberration was no longer detectable. However, new CNAs were identified, including loss of CAE 8p (HSA 8p), loss of CAE 16p (HSA 17p), gain of CAE 16q (HSA 17q), partial loss of CAE 25qdist (HSA 1qdist), and loss of CAE 27 (HSA 4p).

At p31, the gain of CAE 16q and losses of CAE 8p, the partial loss of CAE 25qdist and the loss of CAE 27 remained but were accompanied by several new CNAs. Among these were loss of CAE 3qdist (HSA 13qdist) and CAE 17qmid and an increasingly complex copy-number profile along CAE 8 (HSA 8), with loss of the p-arm now contrasted by a high-level amplification on the q-arm. The peak of the amplification event, spanning a 3.4-Mb region between 101.0 Mb and 104.2 Mb along the CAE 8 chromosome assembly, contains the angioprotein 1 gene (*ANGPT1*), which is involved in regulation of angiogenesis and survival, migration, and proliferation of endothelial cells. Also included in the amplified region is the *MYC* protooncogene at CAE 8: 122 Mb. Additionally, p31 cells exhibited loss of CAE 7p (HSA 4p) and partial deletion along CAE 12 (HSA 9), as well as several low-amplitude CNAs (gains on CAE 1, 2, 4, 11, 13 and 15, and losses of CAE 9q and 17qmid). Notably, at p31, the gain of CAE 21 seen at p11 was also evident. Each of these aberrations was propagated through to p41, with negligible change from the profile observed at p31. In terms of DNA-copy number, the greatest degree of *in vitro* karyotypic evolution occurred between p22 and p31. This was reflected by the total number of discrete CNAs identified in each sample and the overall proportion of the genome residing in regions of CNA relative to the p3 baseline sample (4 CNAs evident at p11, corresponding to 4.7% of the genome, 21 CNAs at p22, 7.2%, 212 CNAs at p31, 26.9% and 247 CNAs at p41, 27.4%).

### miRNA expression profiles/patterns in AGMK1-9T7 cell lines that evolve across the spectrum of neoplastic development

In our earlier studies on the 10–87 VERO cell lines, we had analyzed the miRNA expression profiles as the cells evolved from a non-tumorigenic phenotype at p140 to a tumorigenic phenotype at p190 [13]. This study revealed that four miRNAs (miR-376a, miR-654-3p, miR-543, miR-382) were up-regulated from very low levels in the non-tumorigenic cells to their highest levels in the tumorigenic 10–87 VERO cells at p190 to p256. The association of the high levels of expression of these same miRNAs with the expression of the tumorigenic phenotype by our new line of AGMK cells, the AGMK1-9T7 p40 cells, and the 10–87 VERO cells at p190 or later were confirmed by RT-qPCR [14, 15]. These studies provided additional evidence that these miRNAs might represent biomarkers that could be used to identify tumorigenic AGMK cells.

**Fig 3:**
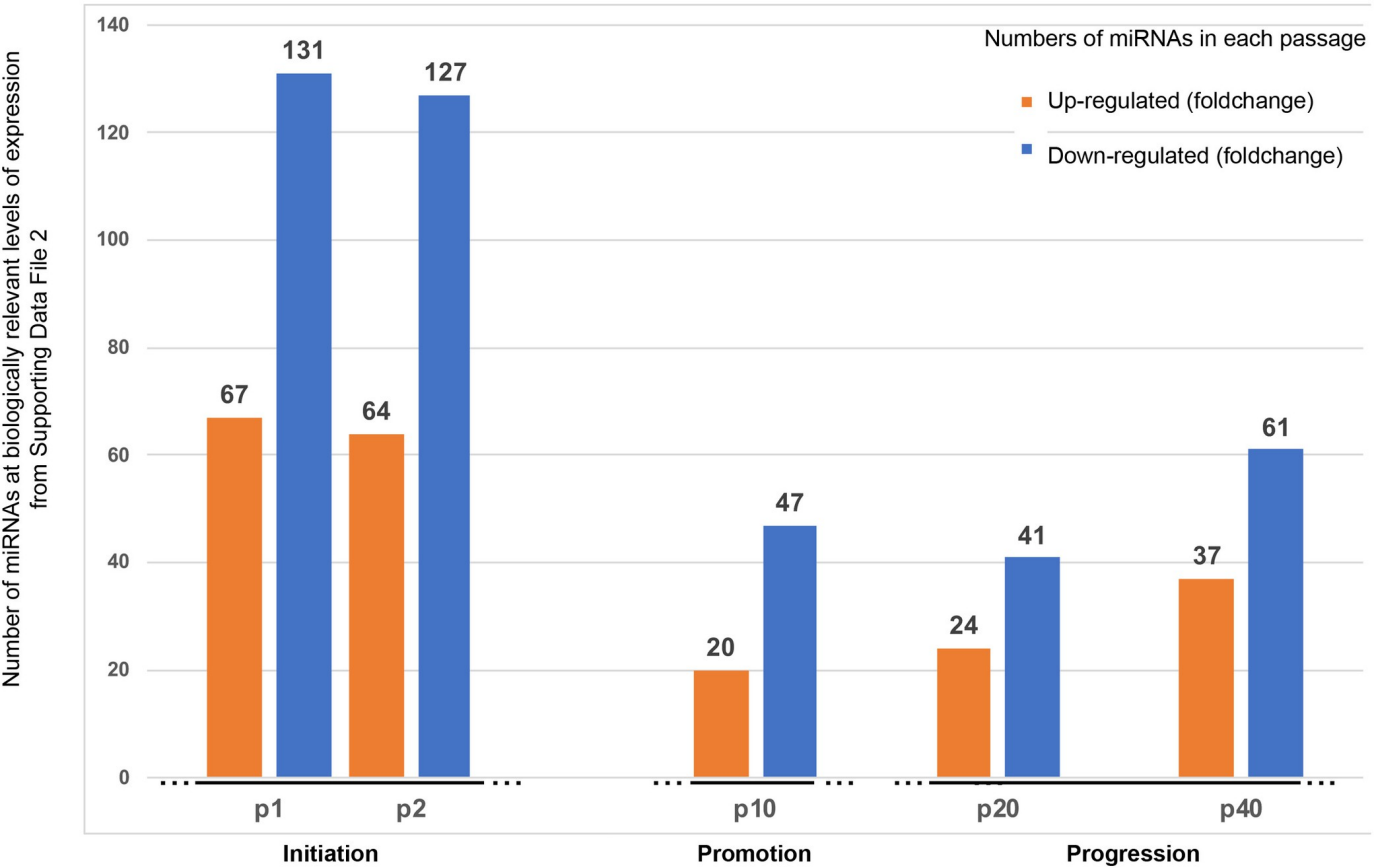
The profiles of BRLE miRNAs expressed across the spectrum of neoplastic development from normal cells to metastatic cancer cells. This figure was designed to show the similarities and differences between the total number of up- and down-regulated miRNAs at the passage levels presented (see Tables 1–5 in S2 Data). To do this, the same levels of activity expressed on the y axis were used for comparison of both up-regulation or down-regulation and were presented as columns of different colors.

**Fig 4:**
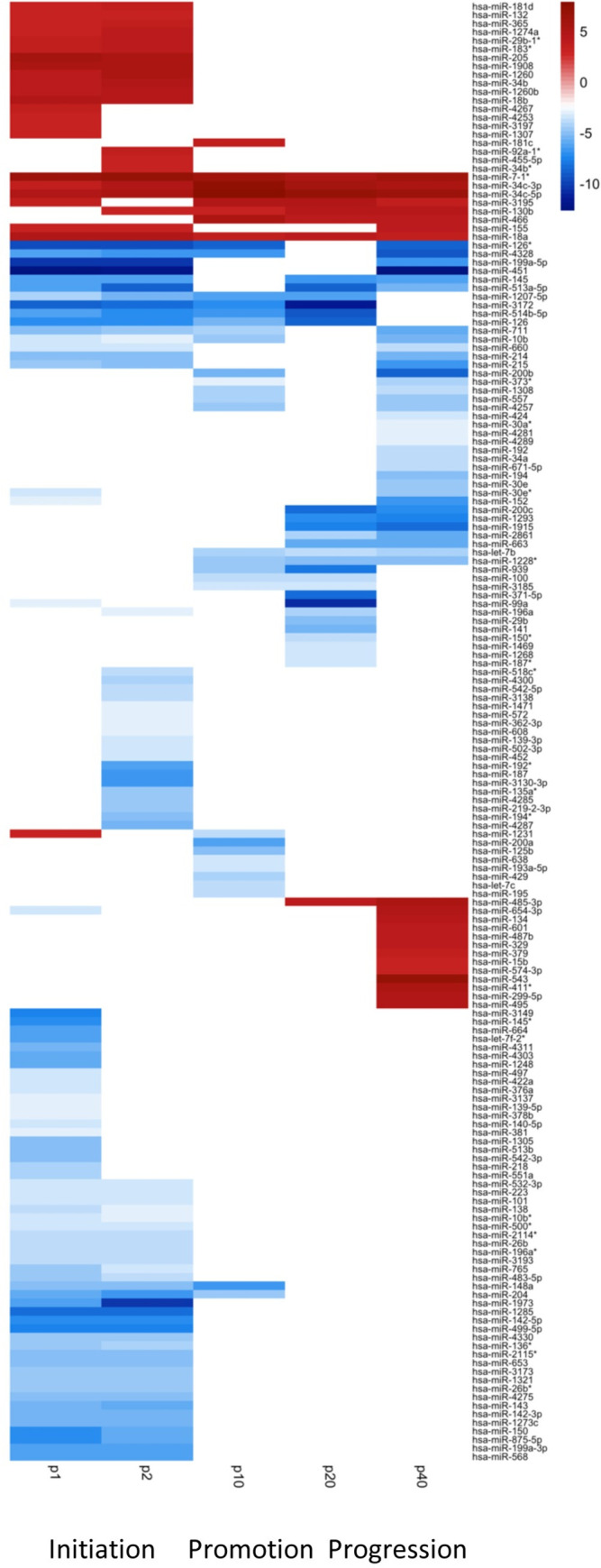
Heatmap of miRNAs from passages p1 to p40. miRNAs from the AGMK1-9T7 cell lines from p1 to p40 that were expressed at BRLE [| log_2_FC | >3 and q < .05] were displayed as a heatmap to provide a graphical representation of changes in miRNA levels across the spectrum of neoplasia.

To further pursue these studies, we examined the profiles of miRNAs that were expressed at BRLE levels in AGMK1-9T7 cells from p0 to p1-p2 and then to p40 to see whether other changes in the expression profiles might be correlated with the neoplastic evolution of the AGMK1-9T7 cell line from its initial establishment in culture to the expression of its tumorigenic phenotype. The data that represent the profiles of miRNA expression are included in Tables 1–5 in S2 Data. To assist in the interpretation of these data, we generated two figures (Figs 3 and 4). Fig 3 summarizes graphically the numbers of up-regulated and down-regulated miRNAs at different passage levels.

Examining the profiles of the expression of these miRNAs across the spectrum of SPNDT by the AGMK1-9T7 cells showed that more miRNAs had altered expression when the cells first grew out and became established in tissue culture at p1 and p2. Following this initial high level of expression, the total numbers of miRNAs expressed declined dramatically by p10. From p10 to p40, the number of miRNAs expressed at significant levels increased from 63 at p10 to 93 at p40. Examining the levels of expression of all of these miRNAs at the various passage levels of the AGMK1-9T7 cell line permitted the development of a complete profile of the expression of these miRNAs across the process of SPNDT in a mammalian species (Fig 3) as well as the generation of a heatmap that depicted these observations (Fig 4).

### Clustering of cell lines based on global miRNA expression

**Fig 5:**
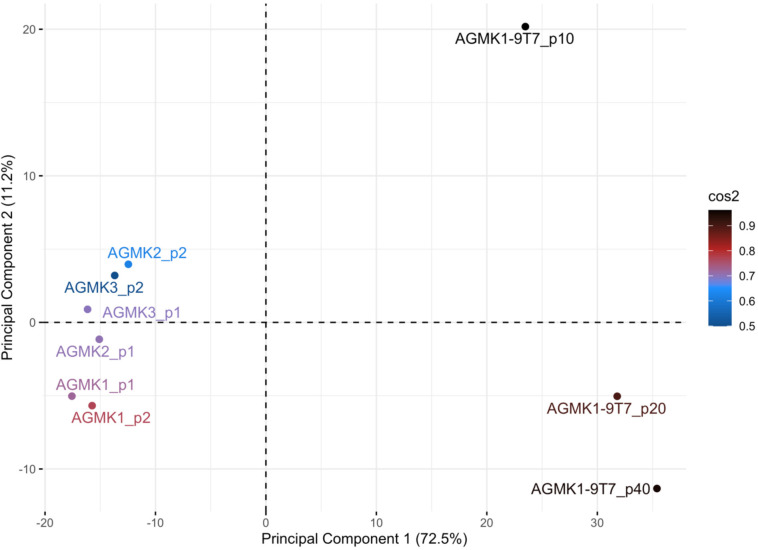
Principal component analysis of cell lines and passages. Cell lines were clustered by passages across global miRNA fold change profiles. Three clusters were observed with p1 and p2 clustered together, p10 separated from the rest of the passages and p20 and p40 clustered together. The squared cosine (cos^2^) represents the contribution of a component to the squared distance of the observation to the origin.

Principal-Component Analysis (PCA) was performed using fold-change values in the expression levels of miRNAs at different passages of the AGMK1-9T7 cell line as the cells evolved to become tumorigenic (see S3 Data). Clustering cell passages by these changes identified three clusters. Passage 2 cells from the kidneys of the two female monkeys (AGMK1, AGMK2) and p1 cells from the one male monkey (AGMK3) formed a distinct cluster that was separate from the other passages (Fig 5). The AGMK1, AGMK2, and monkey cells at p1 and p2 also clustered together, suggesting a different miRNA profile that is distinct from the baseline suspension. Lastly, a third cluster was formed by AGMK1-9T7 cells at p10, p20, and p40; however, p10 was separated from p20 and p40 (Fig 5), p1/p2 were grouped together in Fig 3 and in the PCA, so those were consistent. These clusters can be segregated into individual groups and a pattern outlined by the p1-p2, p10, p20- p40 groups represented the processes involved in SPNDT in tissue culture as described below.

### Comparing SPNDT in vitro and SPNDT in vivo

To examine the possibility that the evolution of AGMK1-9T7 cells to the acquisition of a tumorigenic phenotype could be related to neoplastic processes in vivo in humans and other mammals, four approaches were taken. First, we examined the miRNAs expressed at p1-p2 to p40 that had been reported to be associated with cancers in humans and generated lists revealing these reported associations using the miRCancer Association Database October 2019 and May 2020 (see Tables 1–5 in S2 Data). The number of miRNAs whose activity could be interpreted as BRLE that were expressed at both p1 and p2 represented a total of 389 human and non-human primate miRNAs. However, the expression of non-human primate miRNAs has not been associated with human cancers in the miRCancer Association Database 2019. Therefore, to perform this analysis, the non-human primate miRNAs were omitted when we examined the miRNAs in the miRCancer Association Database. This analysis revealed that 66 of 99 up-regulated hsa-miRNAs at p1, and 57 of 94 of down-regulated hsa-miRNAs at p1, were associated with human cancers. As these data might relate the process of neoplasia in vitro to neoplasia in vivo, we were interested in determining how these hsa miRNAs associated with cancer evolved across the spectrum of neoplasia represented by the various passages of the AGMK1-9T7 cell line. In this analysis, we found that the total numbers of hsa miRNAs (i.e., both up- and down-regulated) associated with human cancers remained relatively unchanged (p1 = 73%, p2 = 69%, p10 = 86%, p20 = s80%, p40 = 71%). The expression profiles of a number of these hsa-miRNAs have been reported in more than 60 studies to be associated with different types of cancers. These miRNAs included: hsa-miR-205 (74 reports), has-miR-155 (85 reports), hsa-miR-21 (561 reports), hsa-miR-218 (68 reports), hsa-miR-145 (138 reports) (from miRCancer Database 4-24-20).

Based on the number of studies that have associated miRNA families and clusters with cancers, our second approach was to generate a list of miRNAs meeting our criteria for BRLE across the spectrum of SPNDT that represented families/clusters that have been associated with neoplastic activity in cancers in animals and humans (Table 1); those miRNAs expressed at BRLE in the AGMK1-9T7 cell lines that were not associated with these family/clusters are shown in Table 2.

**Table 1:** Family/cluster miRNAs expressed in AGMK1-9T7 cell lines.

**A**								
	**Up-Regulated**							
**Families/Clusters**	**miRNA**	**p1**	**p2**		**p10**	**p20**	**p40**	**Count**
**mir-17-92**	**hsa-miR-92a**	**1.53**	**1.72**		**0.84**		1.551	**4**
	**hsa-miR-92b**	**2.17**	**2.41**				1.647	**3**
	**hsa-miR-20a**	**1.75**	**1.76**				0.819	**3**
	**hsa-miR-17**	**1.96**	**1.77**					**2**
	**ggo-miR-17-5p**	**1.90**	**1.79**					**2**
	**hsa-miR-18a**	**4.76**	**4.64**		**3.53**	**4.03**	**1.82**	**5**
	**ggo-miR-18**	**4.80**	**5.03**			**3.30**		**3**
	**hsa-miR-19b**	**1.54**	**1.44**					**2**
	**hsa-miR-19a**	**2.61**	**2.54**					**2**
**mir-106a-93**	**hsa-miR-93**	**2.63**	**2.37**		**2.49**	**2.45**		**4**
	**ggo-miR-93**	**2.65**	**2.44**		**2.52**	**2.50**	**4.34**	**5**
	**hsa-miR-106a**	**1.83**	**1.74**			**1.01**	**4.28**	**4**
	**ggo-miR-106a**	**2.03**	**1.91**				**6.66**	**3**
**mir-106b-25**	**hsa-miR-106b**	**1.95**	**1.86**		**2.15**		1.634	**4**
	**hsa-miR-25**	**2.18**	**2.14**					**2**
**miR-34**	**hsa-miR-34b**	**4.00**	**5.00**					**2**
	**hsa-miR-34c-5p**	**4.63**	**5.37**		**7.91**	**6.97**		**4**
	**hsa-miR-34c-3p**	**3.77**	**4.63**		**7.12**	**5.80**		**4**
	**hsa-miR-34a**	**1.16**	**1.65**		**2.20**			**3**
**miR 23, 24, 27**	hsa-miR-23b*	**1.01**	**1.34**					**2**
**mir-130-301**	**hsa-miR-130b**	**2.87**	**3.09**		**3.30**	**4.25**	**2.49**	**5**
	**hsa-miR-301a**	**1.61**	**1.59**					**2**
**miR-181**	**hsa-miR-181a**				**1.92**	**1.57**	**2.10**	**3**
	**hsa-miR-181b**	**2.00**	**2.17**				**2.01**	**3**
	**ggo-miR-181b**	**1.92**	**1.98**			**1.33**	**5.26**	**4**
	**hsa-miR-181d**	**3.29**	**3.48**					**2**
miR-371-5p, 373*	hsa-miR-373*	**2.69**	**2.56**					**2**
	**total**	**25**	**25**		**10**	**10**	**12**	**26**
**B**								
		**Down-Regulated**						
**Families/Clusters**	**miRNA**	**p1**		**p2**	**p10**	**p20**	**p40**	**Count**
**mir-17-92**	**hsa-miR-19a**						**-2.87**	**1**
**mir-106a-93**	**hsa-miR-93**							**0**
**miR-34**	**hsa-miR-34a**						**-3.91**	**1**
**miR-143-145**	**hsa-miR-145**	**-6.26**		**-6.11**		**-6.80**	**-6.40**	**4**
	**ggo-miR-145**	**-6.64**		**-6.03**			**-6.98**	**3**
	**ggo-miR-143**	**-5.39**		**-5.06**				**2**
	**hsa-miR-143**	**-5.59**		**-5.89**				**2**
**miR-200-429**	**hsa-miR-200c**	**-1.32**		**-1.35**		**-8.26**	**-7.26**	**4**
	**hsa-miR-200a**	**-2.58**		**-2.80**	**-6.47**			**3**
	**hsa-miR-200b**	**-1.50**		**-1.65**	**-5.47**		**-8.68**	**4**
	**ggo-miR-200c**	**-1.15**		**-1.11**	**-9.64**	**-9.64**		**4**
**let-7**	**hsa-let-7f**	**-1.74**		**-1.44**		**-1.85**		**3**
	**hsa-let-7g**	**-1.41**		**-1.12**				**2**
	**hsa-let-7e**	**-1.90**		**-1.54**	**-2.47**	**-2.86**	**-2.96**	**5**
	**hsa-let-7a**	**-1.45**		**-1.17**	**-2.48**	**-2.53**		**4**
	**hsa-let-7d**	**-1.42**		**-1.20**	**-2.58**	**-2.70**		**4**
	**hsa-let-7c**	**-1.87**		**-1.54**	**-3.87**			**3**
	**hsa-let-7b**	**-1.61**		**-1.35**	**-4.29**	**-3.94**	**-4.18**	**5**
miR-371-5p, 373*	**hsa-miR-371-5p**	**-1.29**		**-1.33**		**-8.41**		**3**
	hsa-miR-373*				**-3.09**		**-4.38**	**2**
	**total**	**16**		**16**	**9**	**9**	**9**	

**Table 2:** miRNAs not associated with family/clusters expressed in AGMK1-9T7 cell lines.

**A**										
**Up-regulated non-family miRNAs > 4**										
**miRNA**		**p1**	**miRNA**	**p2**	**miRNA**	**p10**	**miRNA**	**p20**	**miRNA**	**p40**
hsa-miR-7-1*		**6.51**	hsa-miR-7-1*	**7.01**	hsa-miR-7-1*	**5.99**	hsa-miR-7-1*	**5.29**	**has-miR-543**	**6.88**
**hsa-miR-205**		**5.85**	**hsa-miR-205**	**5.23**	**hsa-miR-466**	**5.14**	**hsa-miR-3195**	**5.03**	has-miR-7-1*	**6.25**
**hsa-miR-1908**		**5.24**	**hsa-miR-1260**	**5.12**	**hsa-miR-3195**	**4.89**	**hsa-miR-485-3p**	**4.00**	has-miR—411*	**5.37**
**hsa-miR-18b**		**4.70**	**hsa-miR-1908**	**5.12**					**has-miR—485-3p**	**5.26**
**hsa-miR-1260b**		**4.03**	**ppy-miR-1274A**	**4.83**					**has-miR-654-3p**	**4.94**
			**hsa-miR-18a**	**4.64**					**has-miR-495**	**4.88**
			**hsa-miR-18b**	**4.48**					**has-miR-299-5p**	**4.64**
			**hsa-miR-1260b**	**4.35**					**has-miR-134**	**4.34**
			**hsa-miR-1274a**	**4.16**					**has-miR-134**	**4.28**
			hsa-miR-29b-1*	**4.01**					**has-miR-466**	**4.18**
									**has-miR-155**	**4.17**
									**has-miR-601**	**4.06**
**B**										
	**Down-regulated non-family miRNAs < 4**									
	**miRNA**	**p1**	**miRNA**	**p2**	**miRNA**	**p10**	**miRNA**	**p20**	**miRNA**	**p40**
**1**	**hsa-miR-1285**	**-8.20**	**hsa-miR-451**	**-12.24**	**ggo-miR-200c**	**-9.638**	**ggo-miR-29b**	**-11.58**	**hsa-miR-451**	**-12.66**
**2**	**ppy-miR-1273**	**-7.66**	**hsa-miR-199a-5p**	**-10.39**	**ppa-miR-141**	**-9.626**	**hsa-miR-3172**	**-11.52**	**ggo-miR-141**	**-9.36**
**3**	**hsa-miR-499-5p**	**-7.62**	**hsa-miR-1973**	**-10.19**	**ggo-miR-141**	**-9.364**	**hsa-miR-99a**	**-10.74**	**ggo-miR-10b**	**-9.11**
**4**	**ptr-miR-497**	**-7.34**	**hsa-miR-513a-5p**	**-8.83**	**ggo-miR-10b**	**-7.939**	**ggo-miR-200c**	**-9.64**	**hsa-miR-4328**	**-8.97**
**5**	**hsa-miR-3149**	**-7.32**	**hsa-miR-3172**	**-8.29**	**hsa-miR-3172**	**-6.931**	**ppa-miR-141**	**-9.63**	hsa-miR-126*	**-8.88**
**6**	**ppy-miR-513c**	**-7.29**	**hsa-miR-1285**	**-8.20**	**hsa-miR-514b-5p**	**-6.835**	**hsa-miR-514b-5p**	**-9.30**	**hsa-miR-200b**	**-8.68**
**7**	**hsa-miR-142-5p**	**-7.21**	**ppy-miR-199b-3p**	**-8.15**	**hsa-miR-43281**	**-6.649**	**hsa-miR-513a-5p**	**-8.57**	**hsa-miR-1915**	**-8.17**
**8**	**hsa-miR-150**	**-7.09**	**hsa-miR-499-5p**	**-7.62**	**hsa-miR-148a**	**-6.622**	hsa-miR-126*	**-8.55**	**mml-miR-512-5p**	**-8.09**
**9**	**hsa-miR-875-5p**	**-7.06**	**ppy-miR-513c**	**-7.29**	**hsa-miR-200a**	**-6.465**	**hsa-miR-126**	**-8.55**	**hsa-miR-1293**	**-7.37**
**10**	hsa-let-7f-2*	**-6.44**	**hsa-miR-142-5p**	**-7.21**	**hsa-miR-1207-5p**	**-6.376**	**hsa-miR-371-5p**	**-8.41**	hsa-miR-145*	**-7.29**
**11**	**hsa-miR-1973**	**-6.42**	**hsa-miR-126**	**-7.07**	**ppy-miR-1303**	**-6.302**	**hsa-miR-200c**	**-8.26**	**hsa-miR-200c**	**-7.26**
**12**	**hsa-miR-568**	**-6.32**	hsa-miR-126*	**-7.07**	**hsa-miR-200b**	**-5.468**	**hsa-miR-939**	**-7.86**	**ppy-miR-199b-3p**	**-6.98**
**13**	**hsa-miR-199a-3p**	**-6.20**	**hsa-miR-187**	**-6.62**	**hsa-miR-126**	**-5.429**	**hsa-miR-1915**	**-7.49**	**hsa-miR-152**	**-6.74**
**14**	**hsa-miR-664**	**-6.18**	hsa-miR-187*	**-6.62**	hsa-miR-126*	**-5.429**	**hsa-miR-1293**	**-7.06**	**hsa-miR-215**	**-6.64**
**15**	**ppy-miR-450a**	**-6.18**	**hsa-miR-204**	**-6.60**	**mml-miR-512-5p**	**-5.415**	hsa-miR-145*	**-6.80**	**hsa-miR-199a-5p**	**-6.63**
**16**	**hsa-miR-4303**	**-5.93**	**hsa-miR-4328**	**-6.56**	**hsa-miR-125b**	**-4.919**	**mml-miR-512-5p**	**-6.63**	**hsa-miR-145**	**-6.40**
**17**	**hsa-miR-1248**	**-5.70**	**hsa-miR-3130-3p**	**-6.53**	**hsa-miR-204**	**-4.777**	**hsa-miR-1207-5p**	**-6.34**	**hsa-miR-663**	**-5.94**
**18**	**hsa-miR-1273c**	**-5.62**	**hsa-miR-568**	**-6.32**	**hsa-miR-10b**	**-4.775**	**hsa-miR-663**	**-5.80**	**ppy-miR-1303**	**-5.94**
**19**	**hsa-miR-143**	**-5.59**	**hsa-miR-199a-3p**	**-6.16**	hsa-miR-10b*	**-4.775**	**hsa-miR-141**	**-5.65**	**hsa-miR-2861**	**-5.87**
**20**	**hsa-miR-142-3p**	**-5.47**	**hsa-miR-145**	**-6.11**	**hsa-miR-4257**	**-4.705**	**hsa-miR-29b**	**-5.21**	**hsa-miR-711**	**-5.74**
**21**	**hsa-miR-4311**	**-5.46**	hsa-miR-145*	**-6.11**	**hsa-miR-939**	**-4.533**	hsa-miR-1228*	**-5.13**	**hsa-miR-214**	**-5.59**
**22**	**ggo-miR-143**	**-5.39**	**ggo-miR-145**	**-6.03**	hsa-miR-1228*	**-4.488**	**ggo-miR-214**	**-4.79**	**hsa-miR-513a-5p**	**-5.44**
**23**	**ppy-miR-520c-5p**	**-5.32**	**hsa-miR-150**	**-6.00**	**hsa-miR-1308**	**-4.294**	**hsa-miR-2861**	**-4.33**	**hsa-miR-10b**	**-5.42**
**24**	**ppy-miR-1305**	**-5.26**	**hsa-miR-143**	**-5.89**	**hsa-let-7b**	**-4.291**	**hsa-miR-196a**	**-4.02**	hsa-miR-10b*	**-5.42**
**25**	**hsa-miR-653**	**-5.18**	**hsa-miR-1273c**	**-5.62**	**hsa-miR-711**	**-4.289**	hsa-miR-196a*	**-4.02**	**ppy-miR-298**	**-5.24**
**26**	hsa-miR-2115*	**-5.07**	**hsa-miR-4287**	**-5.61**	**hsa-miR-429**	**-4.192**			hsa-miR-1228*	**-5.03**
**27**	**ppy-miR-1304**	**-5.06**	**hsa-miR-142-3p**	**-5.47**	**hsa-miR-557**	**-4.131**			**ggo-miR-214**	**-4.99**
**28**	**hsa-miR-4275**	**-4.94**	**hsa-miR-1207-5p**	**-5.41**					**hsa-miR-194**	**-4.86**
**29**	**hsa-miR-513b**	**-4.87**	**ppy-miR-520c-5p**	**-5.32**					**hsa-miR-4257**	**-4.77**
**30**	**hsa-miR-542-3p**	**-4.87**	**mml-miR-211**	**-5.26**					**hsa-miR-30e**	**-4.69**
**31**	**hsa-miR-1305**	**-4.84**	**hsa-miR-214**	**-5.23**					hsa-miR-30e*	**-4.69**
**32**	hsa-miR-136*	**-4.78**	**hsa-miR-653**	**-5.18**					**hsa-miR-557**	**-4.65**
**33**	**hsa-miR-1321**	**-4.73**	**pbi-miR-513c**	**-5.08**					**ggo-miR-224**	**-4.46**
**34**	**hsa-miR-3173**	**-4.73**	hsa-miR-2115*	**-5.07**					hsa-miR-373*	**-4.38**
**35**	**ppy-miR-1255b**	**-4.68**	**ggo-miR-143**	**-5.06**					**ppy-miR-1268**	**-4.26**
**36**	**mml-miR-211**	**-4.68**	**ptr-miR-602**	**-5.02**					**hsa-let-7b**	**-4.18**
**37**	**hsa-miR-765**	**-4.64**	**ggo-miR-214**	**-4.99**						
**38**	**hsa-miR-483-5p**	**-4.52**	**hsa-miR-148a**	**-4.95**						
**39**	**hsa-miR-4330**	**-4.47**	**hsa-miR-4275**	**-4.94**						
**40**	**ssy-miR-513c**	**-4.39**	**hsa-miR-215**	**-4.88**						
**41**	**ppy-miR-510**	**-4.37**	**hsa-miR-1321**	**-4.73**						
**42**	**hsa-miR-551a**	**-4.34**	**hsa-miR-3173**	**-4.73**						
**43**	**ppy-miR-616**	**-4.20**	**hsa-miR-4285**	**-4.66**						
**44**	**hsa-miR-218**	**-4.07**	**hsa-miR-219-2-3p**	**-4.65**						
**45**	**ptr-miR-670**	**-4.04**	**mml-miR-604**	**-4.62**						
**46**	hsa-miR-2114*	**-4.00**	**hsa-miR-711**	**-4.61**						
**47**			**hsa-miR-4330**	**-4.47**						
**48**			**ppy-miR-510**	**-4.37**						
**49**			**ptr-miR-661**	**-4.06**						
**50**			**ptr-miR-670**	**-4.04**						
**51**			hsa-miR-2114*	**-4.00**						

We also used this approach to determine which of these miRNAs could be involved in the process of neoplasia during the initial passages in tissue culture. These examples included seven families with a majority of up-regulated miRNAs and four families with a majority of down-regulated miRNAs; some of these miRNAs were both up-regulated and down-regulated at different passage levels, including hsa-miR-19a, hsa-miR-34a, hsa-miR-371-5p. The most active miRNAs expressed in these families/clusters included: up-regulated hsa-miR-18a, ggo-miR-18, hsa-mir-93, hsa-34c-5p, hsa-miR-34a-3p, hsa-130b, and down-regulated hsa-miR-145, ggo-miR-145, hsa-miR-143, ggo-miR-143, hsa-miR-200c, hsa-200a, hsa-200b, ggo-miR-200c and let-7b. From these family/cluster data, there were 26 miRNAs that were up-regulated compared with 17 miRNAs that were down-regulated. The functions reported for these families/clusters miRNAs are summarized in Table 3.

**Table 3:** Functions associated with families/clusters and non-family/cluster miRNAs.

Family/cluster (FC) and non-family/cluster miRNAs involved in WNT-signaling and other pathways		
miRNA	Expression level | log2 FC | >2 and q < 0.05 at passage (p)	miRNA-Regulated pathways and genes
**hsa-miR-148a**	**-4.95 (p2), -6,62 (p10)**	**Hedgehog, Wnt1 tumor-suppressor**
**hsa-miR-152**	**-6.74 (p40)**	**Wnt1 tumor suppressor**
**hsa-miR-199a-3p**	**-6.20 (p1), -6.16 (p2)**	**Hedgehog, FZD7, CD44 tumor suppressor**
**ppy-miR-199b-3p**	**-6.20 (p1), -8.15 (p2), -6.98 (p40)**	**Hedgehog, FDZ7 tumor suppressor**
**hsa-miR-199-5p**	**-10.31**	**Sox-2, NOTCH2, RBPJ, CD133**
**hsa-miR-181a (FC)**	**1.92 (10), 1.57 (p20), 1.54 (p40)**	**WIF-1 tumor suppressor**
**hsa-miR-200b (FC)**	**-5.47 (p10), -8.68 (p40)**	**BM1 tumor suppressor, LIFR**
**hsa-miR-218**	**-4.07 (p1),**	**BM1 tumor suppressor**
**hsa-miR-214**	**-5.23 (p2),**	**β-catenin tumor suppressor**
**ggo-miR-214**	**-4.79 (p20)**	**β-catenin tumor suppressor**
**hsa-miR-106b (FC)**	**1.95 (p1), 1.86 (p2), 2.15 (p10), 1.63 (p40)**	**APC oncogene**
**hsa-miR-155**	**4.17 (p40)**	**APC oncogene**
**hsa-miR-29b**	**5.21 (p20)**	**MMP-2 tumor suppressor**
**ggo-miR-29b**	**11.58 (p20)**	**MMP-2 tumor suppressor**
**hsa-miR-451**	**-12.24 (p2), -12.66 (p40)**	**MMP-9 tumor suppressor**
hsa-miR-34a* (FC)	**1.94 (p1), 2.27 (p2)**	**c-MYC tumor suppressor**
**let-miR-7a (FC)**	**-1.45 (p1), -1.17 (p2), -2.48 (p10), -2.53 (p20)**	**c-MYC tumor suppressor**
**hsa-miR-19a (FC)**	**2.61 (p1), 2.54 (p2)**	**cyclin D1 tumor suppressor**
**hsa-miR-20a**	**1.75 (p1), 1.76 (p2)**	**cyclin D1 tumor suppressor**
**miRNAs in MET**		
**hsa-miR-34a**	**-1.16 (p1), 1.65 (p2), 2.20 (p10)**	**SNAIL1, SNAIL2, ZEB1**
**hsa-miR-34b**	**4.00 (p1), 5.00 (p2)**	** **
**hsa-miR-29b**	**-11.58 (p20)**	**SNAIL**
**let-miR-7b**	**-4.29 (p10), -4.18 (p40)**	**H-RAS, BACH1**
**hsa-miR-204**	**-6.60 (p2), -4.78 (10),**	**SNAIL**
**hsa-miR-30**	**-4.66 (p40)**	**SNAIL**
**ppy-miR-199b-3p**	**-6.20 (p1), -6.16 (p2)**	**FZD7, CD44, tumor suppressor**
**A unique miRNA**		
**hsa-miR-7**	**6.51 (p1), 7.02 (p2), 5.99 (p10), 5.29 (p20), 6.25 (p40)**	**Cir-ITCH/EGFR pathway**

As shown in Table 3, a number of molecular pathways have been reported to be controlled by the miRNAs in the family/clusters and those miRNAs not in the family clusters. Eighteen of the miRNAs expressed in these family/clusters and those expressed individually have been reported to regulate the canonical WNT pathway, the hedgehog pathway, and the genes FDZ7, β-catenin, APC, NLK, MMP-2, MMP-9, c-MYC, CD44, cyclin D1, SOX2, Notch2, RBPJ, and STAT3. These miRNAs include: miR-148a, miR-152, miR-199a-3p, miR-199a-5p, miR-181a, miR-200b, miR-218, miR-214, miR-106b, miR-155, miR-181, miR-29b, miR-451, miR-199a, miR-34a* let-7a, miR-19a, and miR-20a. Of these miRNAs, 8 were up-regulated and 10 were down-regulated in the AGMK1-9T7 cell line model of SPNDT. Several recent articles and reviews describing the complex roles of miRNAs in cancer biology have been published [35–37]. Seven of the miRNAs in this group have been reported to be involved in MET and EMT.

The fourth approach that we used to see how the AGMK1-9T7 tissue-culture model of neoplasia might relate to the process of neoplasia in humans and mammals was an attempt to identify the miRNA expression in neoplasms in these species with the miRNA expression profiles in the AGMK1-9T7 model. Tables 1–5 in S2 Data show that the associations of miRNAs range from 69% to 86% of the AGMK1-9T7 hsa-miRNAs with human cancers. Many studies have confirmed the activity of these miRNAs in a wide variety of cancers in humans and animals [36–38].

Given the role of miRNAs in the AGMK1-9T7 model of neoplasia and in cancers in mammals, we considered the possibility that such data could relate SPNDT in vitro to SPNDT in vivo. In an attempt to examine this relationship, we searched for reports that have associated miRNAs expressed in pre-neoplastic lesions and the evolution of SPNDT in vivo. The initial descriptions of neoplasia in mammals that described neoplastic initiation, promotion and progression were begun in 1934 by Rous and Beard [39]. These types of studies were continued by Berenblum and Shubik [40] and reviewed by Foulds [41].

A number of papers have described the processes of neoplastic initiation, promotion, and progression in human cancers [36, 37, 42–44]. In our review of the papers published on miRNAs associated with these stages of neoplasia in mammalian cancers, we found several papers that described such an association in early stages of colorectal cancers in humans [37, 44]. Maher et al. [36] Kanaan et al. [37] identified six miRNAs (miR-122, miR-181a, miR-146b-5p, let-7e, miR-17, miR-143) that were up-regulated from non-neoplastic tissue to dysplastic tissue; these same miRNAs were down-regulated from dysplasia to cancer. Kanaan et al. [37] also described six miRNAs that affected the p53 pathway (miR-122, miR-214, miR-372, miR-15b, let-7e, miR-17). Of these twelve miRNAs identified by Kanann et al., ten were present in the list of miRNAs expressed by our AGMK1-9T7 cells at p1 and p2 while two were not expressed in the AGMK1-9T7 cells (Table 4). All of these miRNAs described by Kanaan et al. were associated with initiation, promotion, progression in these colorectal tumors. Of these human (hsa) and non-human primate (ggo) miRNAs, the following were up-regulated (hsa-miR-181a, ggo-miR-181a-2, hsa-miR-17, ggo-miR-17-5p, hsa-miR-15b) and down-regulated (hsa-miR-143, ggo-miR-143, hsa-miR-214, ggo-miR-214, let-7e). Marcuello et al. [44] identified six miRNAs that were expressed in advanced adenomas of the colon. Of the six miRNAs described by Marcuello et al. [44], none of these were expressed in our AGMK1-9T7 cells at any of the five passages tested. Maher et al. [36] described 29 miRNAs associated with neoplastic processes in human cancers; of these 29 miRNAs, 22 were expressed in different passages of the AGMK1-9T7 cell line while 7 were not (Table 4). Maher et al. [36] pointed out that these miRNAs *“are regulators of gene expression in all cellular pathways and aberrant miRNA expression is associated with cancer initiation*, *promotion and progression”*. In a chemically-induced neoplasia model in the uterus of Syrian hamsters, seven of the eight miRNAs expressed during neoplastic initiation and promotion were expressed in the AGMK1-9T7 cells (Table 4) [38] These co-expressed miRNAs included miR-200a, miR-200b, miR-200c, miR-29b, miR-429, miR-141, and miR-181a.

**Table 4:** miRNAs expressed in initiation, promotion, progression of human cancers and those expressed by the AGMK1-9T7 model of neoplasia*.

	miRNAs* in human cancers	AGMK1-9T7 p1	AGMK 1-9T7 p2	AGMK1-9T7 p10	AGMK1-9T7 p20	AGMK1-9T7 p40
		Levels of Expression Log2 FC (<0.05)				
1	ggo-miR-31	2.50	2.86	2.63		2.07
2	hsa-miR-31	2.28	2.57	2.57		1.91
3	hsa-miR-20a	1.75	1.76			0.82
4	hsa-miR-184		2.31			
5	hsa-miR-106a	1.83	1.74		1.01	
6	ggo-miR-106a	2.03	1.91			
7	hsa-miR-21	2.25	2.62			2.49
8	hsa-let-7a	-1.45	-1.17	-2.48	-2.53	
9	ggo-miR-145	-6.64	-6.03			-6.98
10	hsa-miR-145	-6.26			-6.80	-6.40
11	hsa-miR-29b-1*	3.53	4.01			
12	ggo-miR-29b	-1.77	-1.98		-11.58	
13	hsa-miR-29b	-1.55			-5.21	
14	hsa-miR-29c*	-2.63	-2.80			
15	hsa-miR-29c	-0.94				
16	mir-519	0				
17	mir-146a	0				
18	hsa-miR-34a	1.16	1.65	2.20		
19	hsa-miR-34a*	1.94	2.27			
20	hsa-miR-34a					-3.91
21	mir-290	0				
22	hsa-miR-378					1.82
23	hsa-miR-378	-1.53				
24	hsa-miR-378b	-3.00				
25	ggo-miR-10b	-4.45	-3.84	-7.94		-9.11
26	hsa-miR-10b	-3.44	-3.04	-4.78		-5.42
27	hsa-miR-10b*	-3.40	-3.10			
28	mir-23a	0				
29	mir-372	0				
30	**mir-122**	0				
31	**mir-146b-5p**	0				
32	hsa-miR-181a-2*	2.83	2.93			
33	**hsa-miR-181a**			1.92	1.57	1.54
34	**ggo-miR-17-5p**	1.90	1.79			
35	**hsa-miR-17**	1.96	1.77		0.99	
36	**hsa-miR-15b**			1.50		3.04
37	**ggo-miR-214**	-4.81	-4.99		-4.79	-4.99
38	**hsa-miR-214**	-5.23	-5.23			-5.59
39	**ggo-miR-143**	-5.39	-5.06			
40	**hsa-miR-143**	-5.59	-5.89			
41	**hsa-let-7e**	-1.90	-1.54	-2.47	-2.86	-2.96

*From Maher et al., [36] bold type.

### Mesenchymal to epithelial transition

As described in our earlier report [14], the AGMK1-9T7 cells undergo mesenchymal-to-epithelial transition (MET) between p36 and p40, which represents a fifth component in this process of neoplasia in these AGMK1-9T7 cells. The MET process and its reverse, epithelial-to-mesenchymal transition (EMT), have been shown to regulate metastasis expressed by a number of tumors [35]. Tumorigenicity studies documented the ability of tumors formed by the AGMK1-9T7 cells at p41 and p44 to metastasize to the lungs of newborn and adult nude mice [14]. These data documented the evolution of the SPNDT process in these cells from normal non-tumorigenic cells to tumorigenic-metastatic cells during serial passage in tissue culture. Several of the miRNAs that were expressed in these cells across SPNDT at BRLE have been reported to be involved the MET/EMT pathways that influence the metastatic process. These miRNAs, their expression levels, and the genes they have been reported to influence in regulating tumor metastasis, are listed in Table 3.

## Discussion

Two procedures were used to examine the evolution of neoplasia in passages of the AGMK1-9T7 cell line established at 10-passage intervals from p1 to p40, when these cells became tumorigenic/metastatic. The first procedure was the evaluation of chromosomal changes across this spectrum of neoplasia, a method that we had used previously to study neoplasia in canine kidney cells [5]. The second procedure was to examine the profiles of miRNA expression in cells of these same cell passages and to compare the expression of these miRNAs with the expression of miRNAs associated with cancer in humans.

### Profiles of DNA-copy-number changes during SPNDT

The AGM karyotype (2n = 60) has a higher chromosome number than most primate genomes, comprising 29 autosomal pairs plus chromosomes X and Y. Since this is due primarily to the occurrence of a small number of ancestral chromosomal fission events in the evolution of the AGM karyotype, a considerable degree of collinearity remains with the human genome [25]. We leveraged this collinearity to use a human microarray for CNA profiling of the AGMK1-9T7 cell line, implementing well-established informatics tools [23] to convert human chromosome coordinates back to the AGM genome assembly. As we have shown previously for other species [24], this molecular recoding approach allows direct interpretation of AGM data in context with the large volume of information accumulated from human studies. The deletions along CAE 12 in AGMK1-9T7 are of note due to identification of a discrete ~9 Mb region of homozygous deletion (CAE 12: 50.0–59.0Mb) in the VERO cell line, flanked by an extensive region of loss of heterozygosity [19]. Using whole-genome next-generation DNA sequencing analysis, this deletion was shown to include the *CDKN2A/B* tumor-suppressor-gene locus and the type I interferon gene cluster, which may contribute to the tumorigenic potential and high susceptibility of VERO cells to viral infection, respectively [19]. Our data show that the deleted regions of VERO and AGMK1-9T7 cells share ~6 Mb in common (CAE 12: 50.6–56.6Mb), within which lies the interferon kappa gene (*IFNK*); however, unlike VERO cells, AGMK1-9T7 cells at p31 and p41 show a normal balanced copy number of *CDKN2A/B* and the interferon gene cluster. Notably, AGMK1-9T7 and VERO cells also share deletions of CAE 7p (which includes *KIT*, *KDR* and *PDGFRA*, CAE 7: 13.4–14.2 Mb), gain of CAE 8qdist (which includes *ANGPT1* and c*MYC*, 102.0 Mb and 122.3 Mb, respectively), and gain of CAE 16q (which includes a cytokine gene cluster), and show evidence of breakage in the WWOX fragile site (CAE 5q: 63.8–64.3Mb). Additional genomic analysis of AGMK1-9T7 cells and comparison with VERO data may identify those molecular events that are more likely to be drivers of key phenotypic behaviors that emerge during cellular evolution *in vitro*, and for elucidating the relative timing of these events.

The apparently transient gain of CAE 21 at p11, followed by its absence at p22 and subsequent reappearance at p31, may reflect fluctuation in the relative contribution of different clones during culture as they emerge, compete, and flourish or recede. It is interesting to note, however, that in our prior studies of karyotype evolution during spontaneous neoplastic development in a novel canine cell line (CKB1-3T7) [5], we identified a transient loss of dog chromosome 27 (CFA 27) at p15, followed by return to a balanced state at p43. This coincided with the appearance of derivative chromosome structures involving CFA 27, which remained evident through to p92. A complete understanding of the genomic processes underlying immortalization and tumorigenicity will require a comprehensive and integrative evaluation of both structural and numerical chromosome alterations, in context with variation at the level of DNA sequence.

### Profiles of miRNA expression in SPNDT

The high level of association of the miRNAs expressed at p1 with cancers in humans and hamsters supported their involvement in the early stages of neoplastic development in tissue culture. Considering these miRNA profiles/patterns of miRNA expression across this spectrum of neoplasia in tissue culture permitted us to hypothesize that the data summarized in Figs 3 and 4 reflected the processes of neoplastic initiation, promotion, and progression that were initially described in animal models and have been reported to reflect neoplasia in humans [45, 46]. Many studies have confirmed the activity of these miRNAs in a wide variety of cancers in humans and animals [36, 42, 45–48]. Given this extensive relationship between miRNAs expressed by the AGMK1-9T7 cells and cancer in vivo, we suggest that such data might indicate a relationship between SPNDT in vitro and SPNDT in vivo.

## Conclusions

In this study, a total of 604 miRNAs, expressed at BRLE at p1, p2, p10, p20, and p40, were detected across the process of SPNDT that occurred during the evolution of normal, non-human primate cells to cells that express a tumorigenic/metastatic phenotype (see S2 Data). We conclude that the net effects of these changes in the miRNA profile across the range of cell-culture passages tested might not just be correlated with the changes in the phenotypes of the cell populations at different passages, including their tumorigenic phenotype, but might be involved in effecting these changes. The AGMK1-9T7 cell line from p1 to p40 represents a cell-culture model that appears to outline the spectrum of neoplasia in vitro. By arranging these miRNAs from their highest to lowest changes at each of these passage levels (Fig 3; S2 Data), they appear to represent the patterns of miRNA expression reflected in animal models of neoplasia (see Fig 3) and patterns more recently detected in human neoplasms [46, 49, 50].

Our studies of AGMK1-9T7 cells at passages p1, p2, p10, p20, p30, and p40, represent a model of SPNDT in tissue culture. Together, these data suggest that the expression of miRNAs is associated with contributions to neoplastic initiation at p1 and p2, while chromosomal aberrations make contributions to neoplastic promotion and progression from p11 to p41. In addition, the 35 additional CNAs between p31 and p41 could be involved with MET which occurred between p36 and p40 [13]. Given the normal cell to tumor/metastatic cell neoplastic processes represented by the AGMK1-9T7 cells, we suggest that models like the AGMK1-9T7 cell line can help provide both a biological and molecular understanding of neoplasia and its contribution to neoplastic disease across multiple species.

## Supporting information

S1 Data Students test using AGMK1-9T7 cell lines. (DOCX)

S2 Data miRNAs in AGMK1-9T7 cells detected in human cancers. (XLSX)

S3 Data Principal components analysis of AGMK1-9T7 cell lines. (XLSX)

S4 Data miRNAs expressed in the kidney cells from 5 different AGMKs. (XLSX)

## Abbreviations

miRNA: MicroRNA
SPNDT: Spontaneous neoplastic development/transformation
AGMK: African green monkey kidney
CAN: DNA copy-number aberrations
CAE: African green monkey chromosome [*Chlorocebus sabaeus*]
BRLE: Expression at biologically relevant levels of activity

## References

[1] Earle WR, Nettleship A (1943). Production of malignancy in vitro. V. Results of injections of cultures into mice *J Natl Cancer I* 4, 213–227.

[2] Gey GO, Coffman W, Kubicek M (1952). Tissue culture studies of the proliferative capacity of cervical carcinoma and normal epithelium. *Cancer Res* 12, 264–265.

[3] Cram LS, Bartholdi MF, Ray FA, Travis GL, Kraemer PM (1983). Spontaneous neoplastic evolution of Chinese hamster cells in culture: multistep progression of karyotype *Cancer Res* 43, 4828–4837. 6883337

[4] Michelhaugh SK, Guastella AR, Varadarajan K, Klinger NV, Parajuli P, Ahmad A, et al. (2015). Development of patient-derived xenograft models from a spontaneously immortal low-grade meningioma cell line, KCI-MENG1 *Journal of Translational Medicine* 13, 227. doi: 10.1186/s12967-015-0596-8 26174772 PMC4501087

[5] Omeir R, Thomas R, Teferedegne B, Williams C, Foseh G, Macauley J, et al. (2015). A novel canine kidney cell line model for the evaluation of neoplastic development: karyotype evolution associated with spontaneous immortalization and tumorigenicity *Chromosome Res* 23, 663–680. doi: 10.1007/s10577-015-9474-8 25957863 PMC4666904

[6] Yasumura Y, Kawakita Y (1963). Study of SV40 in Tissue Culture. *Nippon Rinsho* 21, 1201–1205.

[7] Contreras G, Bather R, Furesz J, Becker BC (1985). Activation of metastatic potential in African green monkey kidney cell lines by prolonged in vitro culture *In Vitro Cell Dev Biol* 21, 649–652. doi: 10.1007/BF02623298 4066602

[8] Furesz J, Fanok A, Contreras G, Becker B (1989). Tumorigenicity testing of various cell substrates for production of biologicals *Dev Biol Stand* 70, 233–243. 2759351

[9] Barrett PN, Portsmouth D, Ehrlich HJ (2013). Vero cell culture-derived pandemic influenza vaccines: preclinical and clinical development *Expert Rev Vaccines* 12, 395–413. doi: 10.1586/erv.13.21 23560920

[10] Betakova T, Svetlikova D, Gocnik M (2013). Overview of measles and mumps vaccine: origin, present, and future of vaccine production *Acta Virol* 57, 91–96. doi: 10.4149/av_2013_02_91 23600866

[11] Montagnon BJ (1989). Polio and rabies vaccines produced in continuous cell lines: a reality for Vero cell line *Dev Biol Stand* 70, 27–47. 2759353

[12] Manohar M, Orrison B, Peden K, Lewis AM Jr. (2008). Assessing the tumorigenic phenotype of VERO cells in adult and newborn nude mice *Biologicals* 36, 65–72. doi: 10.1016/j.biologicals.2007.06.002 17933552

[13] Teferedegne B, Murata H, Quinones M, Peden K, Lewis AM (2010). Patterns of microRNA expression in non-human primate cells correlate with neoplastic development in vitro *PLoS One* 5, e14416. doi: 10.1371/journal.pone.0014416 21203544 PMC3008671

[14] Teferedegne B, Rotroff DM, Macauley J, Foseh G, Lewis G, Motsinger-Rief A, et al. (2017). Assessment of potential miRNA biomarkers of VERO-cell tumorigenicity in a new line (AGMK1-9T7) of African green monkey kidney cells *Vaccine* 35, 5503–5509. doi: 10.1016/j.vaccine.2017.04.004 28434690

[15] Teferedegne B, Macauley J, Foseh G, Dragunsky E, Chumakov K, Murata H, et al. (2014). MicroRNAs as potential biomarkers for VERO cell tumorigenicity *Vaccine* 32, 4799–4805. doi: 10.1016/j.vaccine.2014.05.065 25024114

[16] Formosa A, Markert EK, Lena AM, Italiano D, Finazzi-Agro E, Levine AJ, et al. (2014). MicroRNAs, miR-154, miR-299-5p, miR-376a, miR-376c, miR-377, miR-381, miR-487b, miR-485-3p, miR-495 and miR-654-3p, mapped to the 14q32.31 locus, regulate proliferation, apoptosis, migration and invasion in metastatic prostate cancer cells *Oncogene* 33, 5173–5182. doi: 10.1038/onc.2013.451 24166498

[17] Luk JM, Burchard J, Zhang C, Liu AM, Wong KF, Shek FH, et al. (2011). DLK1-DIO3 genomic imprinted microRNA cluster at 14q32.2 defines a stemlike subtype of hepatocellular carcinoma associated with poor survival *J Biol Chem* 286, 30706–30713. doi: 10.1074/jbc.M111.229831 21737452 PMC3162431

[18] Lin SB, Gregory RI (2015). MicroRNA biogenesis pathways in cancer *Nat Rev Cancer* 15, 321–333. doi: 10.1038/nrc3932 25998712 PMC4859809

[19] Osada N, Kohara A, Yamaji T, Hirayama N, Kasai F, Sekizuka T, et al. (2014). The genome landscape of the african green monkey kidney-derived vero cell line *DNA Res* 21, 673–683. doi: 10.1093/dnares/dsu029 25267831 PMC4263300

[20] Poorman K, Borst L, Moroff S, Roy S, Labelle P, Motsinger-Reif A, et al. (2015). Comparative cytogenetic characterization of primary canine melanocytic lesions using array CGH and fluorescence in situ hybridization *Chromosome Res* 23, 171–186. doi: 10.1007/s10577-014-9444-6 25511566 PMC5462112

[21] Shapiro SG, Raghunath S, Williams C, Motsinger-Reif AA, Cullen JM, Liu T, et al. (2015). Canine urothelial carcinoma: genomically aberrant and comparatively relevant *Chromosome Res* 23, 311–331. doi: 10.1007/s10577-015-9471-y 25783786 PMC4501039

[22] Thomas R, Borst L, Rotroff D, Motsinger-Reif A, Lindblad-Toh K, Modiano JF, et al. (2014). Genomic profiling reveals extensive heterogeneity in somatic DNA copy number aberrations of canine hemangiosarcoma *Chromosome Res* 22, 305–319. doi: 10.1007/s10577-014-9406-z 24599718 PMC5518683

[23] Navarro Gonzalez J, Zweig AS, Speir ML, Schmelter D, Rosenbloom KR, Raney BJ, et al. (2021). The UCSC Genome Browser database: 2021 update *Nucleic Acids Res* 49, D1046–D1057. doi: 10.1093/nar/gkaa1070 33221922 PMC7779060

[24] Thomas R, Seiser EL, Motsinger-Reif A, Borst L, Valli VE, Kelley K, et al. (2011). Refining tumor-associated aneuploidy through ’genomic recoding’ of recurrent DNA copy number aberrations in 150 canine non-Hodgkin lymphomas *Leuk Lymphoma* 52, 1321–1335. doi: 10.3109/10428194.2011.559802 21375435 PMC4304668

[25] Warren WC, Jasinska AJ, Garcia-Perez R, Svardal H, Tomlinson C, Rocchi M, et al. (2015). The genome of the vervet (Chlorocebus aethiops sabaeus) *Genome Res* 25, 1921–1933. doi: 10.1101/gr.192922.115 26377836 PMC4665013

[26] Finelli P, Stanyon R, Plesker R, Ferguson-Smith MA, O’Brien PC, Wienberg J (1999). Reciprocal chromosome painting shows that the great difference in diploid number between human and African green monkey is mostly due to non-Robertsonian fissions *Mamm Genome* 10, 713–718. doi: 10.1007/s003359901077 10384046

[27] Alles J, Fehlmann T, Fischer U, Backes C, Galata V, Minet M, et al. (2019). An estimate of the total number of true human miRNAs *Nucleic Acids Res* 47, 3353–3364. doi: 10.1093/nar/gkz097 30820533 PMC6468295

[28] Brameier M (2010). Genome-wide comparative analysis of microRNAs in three non-human primates *BMC Res Notes* 3, 64. doi: 10.1186/1756-0500-3-64 20214803 PMC2850348

[29] Benjamini Y, Hochberg Y (1995). Controlling the False Discovery Rate—a Practical and Powerful Approach to Multiple Testing *J Roy Stat Soc B Met* 57, 289–300.

[30] Daniels SI, Sille FC, Goldbaum A, Yee B, Key EF, Zhang L, et al. (2014). Improving power to detect changes in blood miRNA expression by accounting for sources of variability in experimental designs *Cancer Epidemiol Biomarkers Prev* 23, 2658–2666. doi: 10.1158/1055-9965.EPI-14-0623 25472674 PMC4256675

[31] Kassambara A (2017). Data Analyses. R package version 1.0.5.

[32] Tang Y HM, Li W (2016). ggfortify: Unified Interface to Visualize Statistical Results of Popular R Packages *The R Journal* 8.2, 474–485.

[33] Angstadt AY, Thayanithy V, Subramanian S, Modiano JF, Breen M (2012). A genome-wide approach to comparative oncology: high-resolution oligonucleotide aCGH of canine and human osteosarcoma pinpoints shared microaberrations *Cancer Genet* 205, 572–587. doi: 10.1016/j.cancergen.2012.09.005 23137772

[34] Italiano A, Thomas R, Breen M, Zhang L, Crago AM, Singer S, et al. (2012). The miR-17-92 cluster and its target THBS1 are differentially expressed in angiosarcomas dependent on MYC amplification *Genes Chromosomes Cancer* 51, 569–578. doi: 10.1002/gcc.21943 22383169 PMC3360479

[35] Nie X, Liu Y, Chen WD, Wang YD (2018). Interplay of miRNAs and Canonical Wnt Signaling Pathway in Hepatocellular Carcinoma *Front Pharmacol* 9, 657. 29977206 10.3389/fphar.2018.00657 PMC6021530

[36] Maher SG, Bibby B, Moddy H, Reid G (2015). *MicroRNAs in Cancer*, *Chapter 4 in Gray*. *Epigenetic Cancer Therapy*, Elsevier Science.

[37] Kanaan Z, Rai SN, Eichenberger MR, Barnes C, Dworkin AM, Weller C, et al. (2012). Differential microRNA expression tracks neoplastic progression in inflammatory bowel disease-associated colorectal cancer *Hum Mutat* 33, 551–560. doi: 10.1002/humu.22021 22241525 PMC4410875

[38] Padmanabhan R, Hendry IR, Knapp JR, Shuai B, Hendry WJ (2017). Altered microRNA expression patterns during the initiation and promotion stages of neonatal diethylstilbestrol-induced dysplasia/neoplasia in the hamster (Mesocricetus auratus) uterus *Cell Biol Toxicol* 33, 483–500. doi: 10.1007/s10565-017-9389-6 28265775 PMC5587358

[39] Rous P, Beard JW (1935). The Progression to Carcinoma of Virus-Induced Rabbit Papillomas (Shope) *J Exp Med* 62, 523–548. doi: 10.1084/jem.62.4.523 19870432 PMC2133298

[40] Berenblum I, Shubik P (1947). The role of croton oil applications, associated with a single painting of a carcinogen, in tumour induction of the mouse’s skin *Br J Cancer* 1, 379–382. doi: 10.1038/bjc.1947.35 18906315 PMC2007538

[41] Foulds L (1969). *Neoplastic Development* *Vol* 1, Vol. 1. Academic Press.

[42] Andersen GB, Knudsen A, Hager H, Hansen LL, Tost J (2018). miRNA profiling identifies deregulated miRNAs associated with osteosarcoma development and time to metastasis in two large cohorts *Mol Oncol* 12, 114–131. doi: 10.1002/1878-0261.12154 29120535 PMC5748490

[43] Calin GA, Croce CM (2007). Chromosomal rearrangements and microRNAs: a new cancer link with clinical implications *J Clin Invest* 117, 2059–2066. doi: 10.1172/JCI32577 17671640 PMC1934569

[44] Marcuello M, Duran-Sanchon S, Moreno L, Lozano JJ, Bujanda L, Castells A, et al. (2019). Analysis of A 6-Mirna Signature in Serum from Colorectal Cancer Screening Participants as Non-Invasive Biomarkers for Advanced Adenoma and Colorectal Cancer Detection *Cancers* 11. doi: 10.3390/cancers11101542 31614785 PMC6827108

[45] Berenblum I, Shubik P (1949). An experimental study of the initiating state of carcinogenesis, and a re-examination of the somatic cell mutation theory of cancer *Br J Cancer* 3, 109–118. doi: 10.1038/bjc.1949.13 18128325 PMC2007559

[46] Scott RE, Wille JJ Jr., Wier ML(1984). Mechanisms for the initiation and promotion of carcinogenesis: a review and a new concept *Mayo Clin Proc* 59, 107–117. doi: 10.1016/s0025-6196(12)60244-4 6366382

[47] Grizzi F, Di Ieva A, Russo C, Frezza EE, Cobos E, Muzzio PC, et al. (2006). Cancer initiation and progression: an unsimplifiable complexity *Theor Biol Med Model* 3, 37. doi: 10.1186/1742-4682-3-37 17044918 PMC1621057

[48] Forterre A, Komuro H, Aminova S, Harada M (2020). A Comprehensive Review of Cancer MicroRNA Therapeutic Delivery Strategies *Cancers (Basel)* 12. doi: 10.3390/cancers12071852 32660045 PMC7408939

[49] Rubin H (2008). Cell-cell contact interactions conditionally determine suppression and selection of the neoplastic phenotype *P Natl Acad Sci USA* 105, 6215–6221. doi: 10.1073/pnas.0800747105 18434545 PMC2359782

[50] Schetter AJ, Leung SY, Sohn JJ, Zanetti KA, Bowman ED, Yanaihara N, et al. (2008). MicroRNA expression profiles associated with prognosis and therapeutic outcome in colon adenocarcinoma *JAMA* 299, 425–436. doi: 10.1001/jama.299.4.425 18230780 PMC2614237

