# Supplementary material for: The AGMK1-9T7 cell model of neoplasia: Evolution of DNA copy-number aberrations and miRNA expression during transition from normal to metastatic cancer cells
Source: PLoS One. 2022 Oct 24;17(10):e0275394. doi: 10.1371/journal.pone.0275394 (PMC9591059; doi:10.1371/journal.pone.0275394)
Supplement: S1 Data — (DOCX) [file pone.0275394.s001.docx]

|  | **Supporting Data File 1. Student’s t-test results to examine miRNA expression**  **Table 1a: AGMK1-9T7 miRNAs whose expression was significantly increased at p1 (q<0.05)** | | | |
| --- | --- | --- | --- | --- |
|  |  |  |  |  |
|  | **miRNA** | **Log2(p1v0 fold change)** | **p1v0 pval** | **p1v0 qval** |
| 1 | hsa-miR-205 | 5.854 | 1.74E-07 | 5.40E-06 |
| 2 | hsa-miR-3141 | 1.816 | 3.35E-07 | 8.10E-06 |
| 3 | hsa-miR-3178 | 2.670 | 3.61E-07 | 8.48E-06 |
| 4 | hsa-miR-1908 | 5.235 | 2.04E-06 | 3.11E-05 |
| 5 | hsa-miR-602 | 2.965 | 2.13E-06 | 3.11E-05 |
| 6 | hsa-miR-17 | 1.960 | 2.16E-06 | 3.11E-05 |
| 7 | hsa-miR-183* | 3.525 | 2.31E-06 | 3.20E-05 |
| 8 | hsa-miR-21 | 2.247 | 2.39E-06 | 3.25E-05 |
| 9 | hsa-miR-3197 | 3.053 | 3.08E-06 | 3.91E-05 |
| 10 | hsa-miR-1231 | 3.181 | 4.48E-06 | 5.26E-05 |
| 11 | hsa-miR-93 | 2.626 | 5.49E-06 | 6.09E-05 |
| 12 | hsa-miR-18b | 4.701 | 5.56E-06 | 6.09E-05 |
| 13 | hsa-miR-106a | 1.834 | 6.68E-06 | 6.91E-05 |
| 14 | hsa-miR-20a | 1.750 | 6.82E-06 | 6.96E-05 |
| 15 | mml-miR-1230 | 3.011 | 1.33E-05 | 0.00010531 |
| 16 | hsa-miR-34c-5p | 4.627 | 1.53E-05 | 0.000118227 |
| 17 | hsa-miR-18a | 4.760 | 1.55E-05 | 0.000118826 |
| 18 | ggo-miR-93 | 2.650 | 1.63E-05 | 0.000122679 |
| 19 | ggo-miR-106a | 2.027 | 1.69E-05 | 0.000125689 |
| 20 | hsa-miR-155 | 3.130 | 2.06E-05 | 0.000145006 |
| 21 | hsa-miR-1226 | 2.488 | 2.21E-05 | 0.000152486 |
| 22 | hsa-miR-34b | 4.000 | 2.31E-05 | 0.00015582 |
| 23 | hsa-miR-718 | 2.043 | 2.36E-05 | 0.000157715 |
| 24 | ggo-miR-17-5p | 1.901 | 2.79E-05 | 0.000177067 |
| 25 | hsa-miR-1538 | 2.501 | 3.21E-05 | 0.000196186 |
| 26 | hsa-miR-4267 | 3.510 | 3.24E-05 | 0.000196264 |
| 27 | hsa-miR-373* | 2.689 | 4.09E-05 | 0.00023306 |
| 28 | ggo-miR-31 | 2.495 | 4.26E-05 | 0.000239061 |
| 29 | hsa-miR-31 | 2.283 | 4.51E-05 | 0.000244508 |
| 30 | hsa-miR-4281 | 1.538 | 4.71E-05 | 0.000249784 |
| 31 | hsa-miR-1280 | 2.042 | 5.13E-05 | 0.000261411 |
| 32 | hsa-miR-181a-2* | 2.827 | 5.19E-05 | 0.000262591 |
| 33 | hsa-miR-1260b | 4.032 | 5.22E-05 | 0.000262591 |
| 34 | hsa-miR-92a | 1.529 | 5.86E-05 | 0.000287463 |
| 35 | hsa-miR-4253 | 3.263 | 7.08E-05 | 0.00033145 |
| 36 | hsa-miR-3195 | 3.521 | 7.10E-05 | 0.00033145 |
| 37 | hsa-miR-25 | 2.183 | 7.35E-05 | 0.000337894 |
| 38 | hsa-miR-933 | 1.802 | 7.37E-05 | 0.000337894 |
| 39 | hsa-miR-762 | 2.494 | 8.59E-05 | 0.000380234 |
| 40 | ggo-miR-18 | 4.797 | 0.000104545 | 0.000435604 |
| 41 | hsa-miR-641 | 1.662 | 0.000110376 | 0.000452728 |
| 42 | ppy-miR-1268 | 1.833 | 0.000131254 | 0.000513746 |
| 43 | hsa-miR-3180-5p | 1.380 | 0.000180949 | 0.00064924 |
| 44 | hsa-miR-671-3p | 1.484 | 0.000191239 | 0.000664621 |
| 45 | hsa-miR-365 | 3.233 | 0.000195972 | 0.000675015 |
| 46 | hsa-miR-455-5p | 2.621 | 0.000263997 | 0.000860453 |
| 47 | hsa-miR-212 | 2.466 | 0.000276362 | 0.00087766 |
| 48 | hsa-miR-1307 | 3.109 | 0.000277454 | 0.00087766 |
| 49 | hsa-miR-181d | 3.291 | 0.000291794 | 0.000911855 |
| 50 | hsa-miR-637 | 2.461 | 0.000295744 | 0.000920489 |
| 51 | hsa-miR-92b | 2.174 | 0.00030149 | 0.000934619 |
| 52 | mml-miR-762 | 2.728 | 0.000303508 | 0.000937127 |
| 53 | hsa-miR-2355 | 1.629 | 0.000315577 | 0.000970525 |
| 54 | hsa-miR-29b-1* | 3.532 | 0.000325372 | 0.000981181 |
| 55 | hsa-miR-132 | 3.311 | 0.000352189 | 0.00104577 |
| 56 | hsa-miR-1469 | 2.618 | 0.000381689 | 0.001107899 |
| 57 | ppy-miR-331-3p | 1.954 | 0.000422046 | 0.001192434 |
| 58 | hsa-miR-34c-3p | 3.771 | 0.000423943 | 0.001192434 |
| 59 | hsa-miR-3185 | 2.339 | 0.000436313 | 0.001218283 |
| 60 | hsa-miR-409-3p | 1.278 | 0.000465803 | 0.001284688 |
| 61 | hsa-miR-23b* | 1.011 | 0.000468135 | 0.001286541 |
| 62 | hsa-miR-106b | 1.949 | 0.00049182 | 0.001342114 |
| 63 | hsa-miR-3124 | 1.983 | 0.000508862 | 0.001374106 |
| 64 | mne-miR-197 | 2.837 | 0.000592261 | 0.001540275 |
| 65 | hsa-miR-191 | 1.063 | 0.000621772 | 0.001600908 |
| 66 | hsa-miR-1238 | 1.800 | 0.000655544 | 0.001671205 |
| 67 | hsa-miR-365* | 1.018 | 0.000661393 | 0.001680588 |
| 68 | hsa-miR-222 | 1.067 | 0.000688998 | 0.001735446 |
| 69 | hsa-miR-34a* | 1.944 | 0.000728721 | 0.001781573 |
| 70 | age-miR-222 | 1.257 | 0.000740515 | 0.001804158 |
| 71 | ppy-miR-298 | 1.935 | 0.000788931 | 0.001880655 |
| 72 | hsa-miR-296-5p | 1.563 | 0.000808001 | 0.001908453 |
| 73 | hsa-miR-1274a | 3.263 | 0.000835466 | 0.001957692 |
| 74 | hsa-miR-106b* | 1.046 | 0.001002962 | 0.002292907 |
| 75 | ppy-miR-1538 | 2.009 | 0.001128269 | 0.002519909 |
| 76 | ppy-miR-1238 | 1.398 | 0.00135948 | 0.002926659 |
| 77 | hsa-miR-20b | 2.416 | 0.001383499 | 0.002948041 |
| 78 | hsa-miR-197 | 2.058 | 0.001456167 | 0.003091861 |
| 79 | hsa-miR-425 | 2.005 | 0.001504477 | 0.00318571 |
| 80 | hsa-miR-28-3p | 1.460 | 0.00157712 | 0.003314965 |
| 81 | hsa-miR-877 | 1.547 | 0.001586539 | 0.003314965 |
| 82 | hsa-miR-130b | 2.866 | 0.001789794 | 0.003698907 |
| 83 | hsa-miR-563 | 1.079 | 0.002099937 | 0.004216196 |
| 84 | hsa-miR-183 | 2.164 | 0.002282135 | 0.004500392 |
| 85 | hsa-miR-19b | 1.535 | 0.002392649 | 0.004670788 |
| 86 | hsa-miR-19a | 2.612 | 0.002480192 | 0.004829519 |
| 87 | hsa-miR-193a-5p | 1.612 | 0.002553192 | 0.00494681 |
| 88 | hsa-miR-2861 | 1.182 | 0.002680587 | 0.005167799 |
| 89 | ggo-miR-181b | 1.921 | 0.002828946 | 0.005373611 |
| 90 | hsa-miR-182 | 1.857 | 0.003122771 | 0.005766035 |
| 91 | ggo-miR-20 | 1.710 | 0.003195109 | 0.005881733 |
| 92 | ptr-miR-25 | 2.166 | 0.0032858 | 0.006034348 |
| 93 | hsa-miR-1228* | 1.569 | 0.003414544 | 0.006231145 |
| 94 | hsa-miR-455-3p | 2.118 | 0.00342512 | 0.006231145 |
| 95 | ppy-miR-602 | 2.923 | 0.003513081 | 0.006346475 |
| 96 | hsa-miR-7-1* | 6.512 | 0.003642448 | 0.006549646 |
| 97 | ptr-miR-1236 | 1.235 | 0.003762743 | 0.006719185 |
| 98 | ppy-miR-1181 | 1.987 | 0.004086643 | 0.007214461 |
| 99 | hsa-miR-24-2* | 2.359 | 0.004143272 | 0.00728126 |
| 100 | ggo-miR-183 | 2.310 | 0.004665664 | 0.008077836 |
| 101 | hsa-miR-181b | 2.004 | 0.004850795 | 0.008298821 |
| 102 | hsa-miR-340* | 1.860 | 0.005298466 | 0.008830777 |
| 103 | mml-miR-1235 | 1.809 | 0.005363778 | 0.00887027 |
| 104 | hsa-miR-221* | 1.097 | 0.006115076 | 0.009935396 |
| 105 | hsa-miR-1275 | 1.433 | 0.006906827 | 0.010968834 |
| 106 | hsa-miR-301a | 1.607 | 0.006934216 | 0.01098981 |
| 107 | hsa-miR-93* | 1.819 | 0.007490214 | 0.011774678 |
| 108 | hsa-miR-330-3p | 1.290 | 0.009819583 | 0.014863626 |
| 109 | hsa-miR-331-3p | 1.357 | 0.010365557 | 0.015482398 |
| 110 | hsa-miR-4290 | 1.607 | 0.010480111 | 0.015619396 |
| 111 | ppy-miR-182 | 1.424 | 0.011361803 | 0.016836324 |
| 112 | hsa-miR-1260 | 3.807 | 0.012029291 | 0.017690134 |
| 113 | hsa-miR-421 | 1.591 | 0.013163945 | 0.018962931 |
| 114 | hsa-miR-4321 | 1.464 | 0.013275527 | 0.019079139 |
| 115 | ptr-miR-582 | 1.693 | 0.014702529 | 0.020868975 |
| 116 | hsa-miR-4258 | 1.244 | 0.01968266 | 0.027239396 |
| 117 | hsa-miR-34a | 1.164 | 0.021391522 | 0.029358052 |
| 118 | hsa-miR-1181 | 2.763 | 0.021402967 | 0.029358052 |
| 119 | hsa-miR-550 | 1.499 | 0.025510764 | 0.03456441 |
| 120 | hsa-miR-320d | 1.120 | 0.027472404 | 0.036899676 |
| 121 | hsa-miR-598 | 1.380 | 0.034442605 | 0.044937742 |
| 122 | hsa-miR-320a | 1.089 | 0.034687874 | 0.045181685 |
| 123 | ppy-miR-1274a | 3.478 | 0.037091724 | 0.048150898 |

|  | **Table 1b: AGMK1-9T7 miRNAs whose expression was significantly decreased at p1 (q<0.05)** | | | |
| --- | --- | --- | --- | --- |
|  |  |  |  |  |
|  | **miRNA** | **Log2(p1v0 fold change)** | **p1v0 pval** | **p1v0 qval** |
| 1 | hsa-miR-451 | -12.655 | 6.11E-13 | 3.66E-10 |
| 2 | hsa-miR-199a-5p | -10.385 | 1.69E-11 | 3.28E-09 |
| 3 | ppy-miR-1303 | -9.109 | 2.58E-09 | 2.77E-07 |
| 4 | hsa-miR-1248 | -5.700 | 3.16E-09 | 2.77E-07 |
| 5 | hsa-miR-150 | -7.094 | 3.22E-09 | 2.77E-07 |
| 6 | hsa-miR-664 | -6.183 | 1.47E-08 | 1.04E-06 |
| 7 | hsa-miR-513a-5p | -6.455 | 1.08E-07 | 4.59E-06 |
| 8 | hsa-miR-145* | -7.290 | 1.12E-07 | 4.59E-06 |
| 9 | ppy-miR-1273 | -7.656 | 1.31E-07 | 4.59E-06 |
| 10 | ggo-miR-145 | -6.640 | 1.35E-07 | 4.59E-06 |
| 11 | hsa-miR-568 | -6.320 | 1.36E-07 | 4.59E-06 |
| 12 | hsa-miR-142-3p | -5.470 | 1.93E-07 | 5.54E-06 |
| 13 | hsa-miR-499-5p | -7.623 | 3.08E-07 | 7.71E-06 |
| 14 | hsa-miR-1285 | -8.201 | 5.64E-07 | 1.18E-05 |
| 15 | hsa-miR-10b* | -3.395 | 8.00E-07 | 1.59E-05 |
| 16 | hsa-miR-126 | -7.270 | 8.18E-07 | 1.59E-05 |
| 17 | hsa-miR-142-5p | -7.206 | 1.37E-06 | 2.46E-05 |
| 18 | hsa-miR-152 | -3.011 | 1.56E-06 | 2.68E-05 |
| 19 | hsa-miR-1273c | -5.620 | 2.09E-06 | 3.11E-05 |
| 20 | hsa-miR-143 | -5.593 | 2.57E-06 | 3.43E-05 |
| 21 | hsa-miR-1305 | -4.841 | 2.99E-06 | 3.86E-05 |
| 22 | hsa-miR-3149 | -7.317 | 6.14E-06 | 6.52E-05 |
| 23 | hsa-miR-203 | -2.307 | 6.47E-06 | 6.78E-05 |
| 24 | ppy-miR-1305 | -5.261 | 7.57E-06 | 7.58E-05 |
| 25 | hsa-miR-1975 | -1.952 | 7.74E-06 | 7.59E-05 |
| 26 | hsa-miR-513b | -4.874 | 8.46E-06 | 8.19E-05 |
| 27 | ppy-miR-1255b | -4.682 | 8.87E-06 | 8.39E-05 |
| 28 | hsa-miR-199a-3p | -6.196 | 9.17E-06 | 8.56E-05 |
| 29 | hsa-miR-26a | -1.718 | 9.32E-06 | 8.60E-05 |
| 30 | hsa-miR-4311 | -5.459 | 1.14E-05 | 0.000102071 |
| 31 | hsa-miR-126* | -9.465 | 1.25E-05 | 0.000104537 |
| 32 | ppy-miR-520c-5p | -5.316 | 1.29E-05 | 0.000104537 |
| 33 | hsa-miR-145 | -6.262 | 1.30E-05 | 0.000104537 |
| 34 | hsa-miR-497 | -3.533 | 1.86E-05 | 0.000135879 |
| 35 | hsa-miR-136* | -4.781 | 2.02E-05 | 0.000143353 |
| 36 | ggo-miR-30b | -1.527 | 2.22E-05 | 0.000152486 |
| 37 | hsa-miR-1207-5p | -4.409 | 2.49E-05 | 0.000163658 |
| 38 | hsa-miR-4303 | -5.927 | 3.19E-05 | 0.000196159 |
| 39 | hsa-miR-551a | -4.343 | 3.36E-05 | 0.000200394 |
| 40 | hsa-miR-653 | -5.177 | 3.57E-05 | 0.000208081 |
| 41 | hsa-miR-875-5p | -7.055 | 3.65E-05 | 0.000210894 |
| 42 | hsa-miR-542-3p | -4.874 | 4.41E-05 | 0.000244193 |
| 43 | ppy-miR-450a | -6.180 | 4.48E-05 | 0.000244508 |
| 44 | hsa-miR-148a | -4.896 | 4.78E-05 | 0.000252117 |
| 45 | ppy-miR-1304 | -5.059 | 4.97E-05 | 0.000259934 |
| 46 | hsa-let-7f-2* | -6.443 | 5.01E-05 | 0.000259934 |
| 47 | hsa-miR-204 | -6.050 | 6.09E-05 | 0.000296836 |
| 48 | hsa-miR-502-3p | -2.617 | 6.32E-05 | 0.000304116 |
| 49 | ggo-miR-143 | -5.386 | 6.64E-05 | 0.000317453 |
| 50 | hsa-miR-99a | -3.114 | 7.31E-05 | 0.000337894 |
| 51 | hsa-miR-1246 | -1.779 | 8.35E-05 | 0.000374143 |
| 52 | hsa-miR-362-3p | -2.966 | 9.57E-05 | 0.000411712 |
| 53 | hsa-miR-26b* | -4.833 | 0.000102034 | 0.000430272 |
| 54 | ppy-miR-616 | -4.196 | 0.00010271 | 0.000430272 |
| 55 | ppy-miR-513c | -7.292 | 0.000114771 | 0.000460866 |
| 56 | hsa-miR-138 | -3.729 | 0.000124375 | 0.000491787 |
| 57 | hsa-miR-140-5p | -3.243 | 0.000126811 | 0.000498876 |
| 58 | ppa-miR-141 | -2.331 | 0.000137693 | 0.00053154 |
| 59 | hsa-miR-214 | -5.227 | 0.000167725 | 0.000613146 |
| 60 | hsa-miR-98 | -2.688 | 0.000173469 | 0.000631165 |
| 61 | mml-miR-512-5p | -3.010 | 0.000174402 | 0.000631597 |
| 62 | hsa-miR-223 | -3.538 | 0.000180022 | 0.000648916 |
| 63 | hsa-miR-500 | -1.898 | 0.000182835 | 0.000652982 |
| 64 | hsa-miR-101 | -3.535 | 0.000190947 | 0.000664621 |
| 65 | hsa-miR-125b-2* | -1.581 | 0.000199953 | 0.000685678 |
| 66 | hsa-miR-2115* | -5.066 | 0.000231892 | 0.000777991 |
| 67 | ssy-miR-513c | -4.392 | 0.000245215 | 0.00081563 |
| 68 | hsa-miR-4275 | -4.939 | 0.000264242 | 0.000860453 |
| 69 | mml-miR-297 | -3.369 | 0.000267378 | 0.000863408 |
| 70 | hsa-miR-500b | -2.350 | 0.00027656 | 0.00087766 |
| 71 | hsa-miR-660 | -3.267 | 0.000284503 | 0.000896301 |
| 72 | hsa-miR-377 | -2.437 | 0.000289542 | 0.000908483 |
| 73 | hsa-miR-196a | -2.606 | 0.000318701 | 0.000972413 |
| 74 | hsa-miR-483-5p | -4.519 | 0.000321312 | 0.000972912 |
| 75 | hsa-let-7f | -1.736 | 0.000321375 | 0.000972912 |
| 76 | ggo-miR-10b | -4.450 | 0.000349336 | 0.001041291 |
| 77 | ggo-miR-101 | -2.843 | 0.00036863 | 0.001086266 |
| 78 | hsa-miR-30b | -1.637 | 0.000375442 | 0.001097992 |
| 79 | hsa-miR-3193 | -3.773 | 0.000402089 | 0.001149886 |
| 80 | hsa-miR-381 | -3.123 | 0.000418128 | 0.001186993 |
| 81 | hsa-miR-215 | -4.636 | 0.00043701 | 0.001218283 |
| 82 | hsa-miR-30a | -1.397 | 0.000450564 | 0.001248037 |
| 83 | hsa-miR-30c | -1.330 | 0.000450904 | 0.001248037 |
| 84 | hsa-miR-196b* | -2.361 | 0.000486235 | 0.001331563 |
| 85 | mml-miR-765 | -2.856 | 0.000550502 | 0.001461012 |
| 86 | ggo-miR-141 | -1.673 | 0.000560837 | 0.001478397 |
| 87 | hsa-miR-10b | -3.443 | 0.000607882 | 0.001575614 |
| 88 | hsa-miR-26b | -3.815 | 0.000671477 | 0.001700635 |
| 89 | ppy-miR-510 | -4.369 | 0.000702408 | 0.001735446 |
| 90 | hsa-miR-218 | -4.073 | 0.000703135 | 0.001735446 |
| 91 | hsa-miR-422a | -3.374 | 0.000742615 | 0.001804158 |
| 92 | hsa-miR-514b-5p | -6.125 | 0.00077793 | 0.001877965 |
| 93 | lla-miR-198 | -3.653 | 0.000790008 | 0.001880655 |
| 94 | hsa-miR-572 | -2.232 | 0.000791088 | 0.001880655 |
| 95 | age-miR-198 | -2.365 | 0.000807014 | 0.001908453 |
| 96 | hsa-let-7a | -1.448 | 0.001090299 | 0.002470707 |
| 97 | hsa-miR-200a | -2.583 | 0.00109919 | 0.002475282 |
| 98 | hsa-miR-532-5p | -2.746 | 0.001103967 | 0.002475282 |
| 99 | hsa-miR-500* | -3.227 | 0.001104324 | 0.002475282 |
| 100 | ggo-miR-214 | -4.806 | 0.001157436 | 0.002562895 |
| 101 | hsa-miR-29c* | -2.626 | 0.001240515 | 0.002723511 |
| 102 | hsa-miR-1973 | -6.418 | 0.001384628 | 0.002948041 |
| 103 | hsa-miR-886-3p | -2.946 | 0.001547185 | 0.003267217 |
| 104 | ppy-miR-10a | -2.303 | 0.001579403 | 0.003314965 |
| 105 | hsa-miR-489 | -2.120 | 0.001714043 | 0.003570922 |
| 106 | hsa-miR-30d | -1.364 | 0.001878122 | 0.003836379 |
| 107 | hsa-miR-3173 | -4.728 | 0.001878801 | 0.003836379 |
| 108 | ptr-miR-764 | -2.836 | 0.002052588 | 0.004142594 |
| 109 | hsa-miR-378b | -3.002 | 0.002156048 | 0.004317667 |
| 110 | hsa-miR-30a* | -2.343 | 0.002196272 | 0.004386884 |
| 111 | hsa-miR-10a | -2.374 | 0.002211482 | 0.004405908 |
| 112 | ppy-miR-199b-3p | -9.515 | 0.002260188 | 0.004479913 |
| 113 | hsa-let-7d | -1.422 | 0.002540078 | 0.004933735 |
| 114 | hsa-miR-574-5p | -2.850 | 0.002595514 | 0.005016268 |
| 115 | hsa-miR-575 | -2.858 | 0.002721077 | 0.005221195 |
| 116 | ppa-miR-188 | -1.546 | 0.002755444 | 0.0052657 |
| 117 | hsa-miR-765 | -4.644 | 0.002758547 | 0.0052657 |
| 118 | hsa-miR-30e | -2.013 | 0.003041976 | 0.005680799 |
| 119 | hsa-miR-30e* | -3.493 | 0.003096594 | 0.005741292 |
| 120 | hsa-miR-424 | -2.341 | 0.003124819 | 0.005766035 |
| 121 | hsa-let-7c | -1.869 | 0.003461541 | 0.006282657 |
| 122 | hsa-let-7g | -1.407 | 0.00369543 | 0.006629533 |
| 123 | hsa-let-7e | -1.896 | 0.00384442 | 0.006849254 |
| 124 | hsa-miR-362-5p | -2.011 | 0.004134195 | 0.00728126 |
| 125 | hsa-miR-135a* | -1.635 | 0.004535877 | 0.007899561 |
| 126 | hsa-miR-192 | -2.303 | 0.004724969 | 0.00815557 |
| 127 | hsa-miR-376a | -3.344 | 0.004814697 | 0.008255288 |
| 128 | hsa-miR-141 | -1.636 | 0.005030338 | 0.008568158 |
| 129 | ppy-miR-1246 | -1.713 | 0.005140621 | 0.008736802 |
| 130 | ptr-miR-670 | -4.044 | 0.005201718 | 0.008759201 |
| 131 | hsa-miR-4306 | -1.670 | 0.005221614 | 0.008759201 |
| 132 | hsa-miR-361-5p | -1.628 | 0.005313082 | 0.008836134 |
| 133 | mml-miR-211 | -4.676 | 0.005632014 | 0.009267114 |
| 134 | hsa-miR-194 | -1.815 | 0.005747895 | 0.009437752 |
| 135 | hsa-miR-125a-5p | -1.252 | 0.006305686 | 0.010223653 |
| 136 | hsa-miR-2114* | -4.000 | 0.006375348 | 0.01029353 |
| 137 | hsa-miR-4330 | -4.470 | 0.006414 | 0.010312967 |
| 138 | hsa-miR-1321 | -4.728 | 0.006617963 | 0.010575095 |
| 139 | hsa-miR-187* | -2.425 | 0.006755892 | 0.010773285 |
| 140 | ggo-miR-223 | -1.698 | 0.006784127 | 0.010796096 |
| 141 | hsa-miR-196a* | -3.874 | 0.007433854 | 0.011709832 |
| 142 | hsa-let-7b | -1.608 | 0.007972777 | 0.0124324 |
| 143 | hsa-miR-148b | -1.831 | 0.008810629 | 0.013575026 |
| 144 | mml-miR-936 | -1.359 | 0.009050717 | 0.013889714 |
| 145 | hsa-miR-186 | -1.998 | 0.009697055 | 0.014735721 |
| 146 | hsa-miR-1972 | -1.270 | 0.009723388 | 0.014746821 |
| 147 | hsa-miR-200b | -1.497 | 0.009841409 | 0.014867626 |
| 148 | hsa-miR-3177 | -0.933 | 0.010260542 | 0.015410053 |
| 149 | hsa-miR-195 | -2.346 | 0.010949216 | 0.01625602 |
| 150 | ptr-miR-497 | -7.338 | 0.011388672 | 0.016843933 |
| 151 | hsa-miR-532-3p | -3.381 | 0.011522425 | 0.017009294 |
| 152 | hsa-miR-125b | -1.304 | 0.012118757 | 0.017787948 |
| 153 | ppy-miR-1254 | -2.588 | 0.012247679 | 0.017943197 |
| 154 | hsa-miR-200c | -1.317 | 0.012458458 | 0.018214221 |
| 155 | ggo-miR-29b | -1.768 | 0.012743537 | 0.018555023 |
| 156 | hsa-miR-3172 | -8.641 | 0.012916612 | 0.01871098 |
| 157 | hsa-miR-374b | -2.325 | 0.012988213 | 0.018779599 |
| 158 | hsa-miR-4298 | -1.375 | 0.013293851 | 0.019079139 |
| 159 | hsa-miR-4328 | -6.308 | 0.014127064 | 0.020162936 |
| 160 | hsa-miR-3194 | -1.799 | 0.014629748 | 0.02080377 |
| 161 | ggo-miR-186 | -2.374 | 0.015307353 | 0.021648173 |
| 162 | hsa-miR-378 | -1.532 | 0.015672671 | 0.022081787 |
| 163 | hsa-miR-376c | -1.834 | 0.015948854 | 0.022351467 |
| 164 | ggo-miR-200c | -1.146 | 0.016579177 | 0.023151104 |
| 165 | hsa-miR-371-5p | -1.294 | 0.017816557 | 0.02483423 |
| 166 | hsa-miR-4324 | -1.761 | 0.019735284 | 0.027263539 |
| 167 | hsa-miR-124 | -0.892 | 0.020891691 | 0.028758545 |
| 168 | hsa-miR-3137 | -3.054 | 0.022943376 | 0.031359994 |
| 169 | hsa-miR-4289 | -2.503 | 0.023789007 | 0.032458592 |
| 170 | hsa-miR-29c | -0.943 | 0.024598166 | 0.033386303 |
| 171 | hsa-miR-139-5p | -3.014 | 0.02605748 | 0.035120951 |
| 172 | hsa-miR-429 | -1.263 | 0.027230356 | 0.036638065 |
| 173 | hsa-miR-654-3p | -3.447 | 0.028506612 | 0.038156518 |
| 174 | hsa-miR-608 | -1.119 | 0.03204377 | 0.042306511 |
| 175 | hsa-miR-29b | -1.547 | 0.032919525 | 0.043232972 |
| 176 | hsa-miR-486-5p | -2.668 | 0.032968627 | 0.043232972 |
| 177 | ggo-miR-187 | -2.987 | 0.033788952 | 0.044159254 |
| 178 | hsa-miR-711 | -5.137 | 0.035271925 | 0.045865339 |
| 179 | hsa-miR-4314 | -1.946 | 0.037272971 | 0.048305272 |

|  | **Table 2a: AGMK1-9T7 miRNAs whose expression was significantly increased at p2 (q<0.05)** | | | |
| --- | --- | --- | --- | --- |
|  |  |  |  |  |
|  | **miRNA** | **Log2(p2v0 fold change)** | **p2v0 pval** | **p2v0 qval** |
| 1 | hsa-miR-21 | 2.624 | 1.30E-10 | 2.02E-08 |
| 2 | ggo-miR-18 | 5.034 | 8.66E-08 | 4.24E-06 |
| 3 | hsa-miR-877 | 1.797 | 1.71E-07 | 5.40E-06 |
| 4 | hsa-miR-205 | 5.227 | 1.10E-06 | 2.08E-05 |
| 5 | hsa-miR-340* | 2.158 | 1.40E-06 | 2.47E-05 |
| 6 | hsa-miR-183* | 3.877 | 1.71E-06 | 2.82E-05 |
| 7 | hsa-miR-3178 | 2.499 | 1.71E-06 | 2.82E-05 |
| 8 | hsa-miR-7-1* | 7.009 | 1.81E-06 | 2.93E-05 |
| 9 | hsa-miR-1908 | 5.121 | 2.26E-06 | 3.18E-05 |
| 10 | hsa-miR-3141 | 1.617 | 2.78E-06 | 3.65E-05 |
| 11 | hsa-miR-181a-2* | 2.930 | 3.39E-06 | 4.17E-05 |
| 12 | hsa-miR-34b | 5.000 | 4.29E-06 | 5.11E-05 |
| 13 | ptr-miR-582 | 1.747 | 5.08E-06 | 5.79E-05 |
| 14 | hsa-miR-1231 | 2.245 | 1.06E-05 | 9.67E-05 |
| 15 | hsa-miR-18b | 4.483 | 1.31E-05 | 0.000104537 |
| 16 | ggo-miR-31 | 2.858 | 1.31E-05 | 0.000104537 |
| 17 | hsa-miR-4267 | 2.967 | 1.58E-05 | 0.000120386 |
| 18 | hsa-miR-31 | 2.567 | 2.00E-05 | 0.000143353 |
| 19 | hsa-miR-3197 | 2.127 | 2.08E-05 | 0.00014515 |
| 20 | hsa-miR-20a | 1.761 | 2.26E-05 | 0.000153697 |
| 21 | hsa-miR-598 | 1.604 | 2.48E-05 | 0.000163658 |
| 22 | mml-miR-1230 | 2.606 | 2.56E-05 | 0.000166111 |
| 23 | hsa-miR-1260b | 4.353 | 2.57E-05 | 0.000166111 |
| 24 | hsa-miR-34c-5p | 5.368 | 2.66E-05 | 0.000170055 |
| 25 | hsa-miR-197 | 2.185 | 3.00E-05 | 0.00018874 |
| 26 | hsa-miR-718 | 1.934 | 3.14E-05 | 0.000195247 |
| 27 | hsa-miR-92a | 1.720 | 3.15E-05 | 0.000195247 |
| 28 | hsa-miR-155 | 3.089 | 3.45E-05 | 0.000204286 |
| 29 | ppy-miR-1274a | 4.829 | 3.81E-05 | 0.000218487 |
| 30 | hsa-miR-93 | 2.374 | 4.26E-05 | 0.000239061 |
| 31 | hsa-miR-17 | 1.765 | 4.57E-05 | 0.000244508 |
| 32 | hsa-miR-18a | 4.640 | 5.11E-05 | 0.000261411 |
| 33 | hsa-miR-1280 | 2.221 | 5.62E-05 | 0.000281067 |
| 34 | hsa-miR-106a | 1.741 | 5.77E-05 | 0.000286733 |
| 35 | ggo-miR-93 | 2.442 | 6.78E-05 | 0.000322571 |
| 36 | hsa-miR-762 | 2.411 | 8.55E-05 | 0.000380234 |
| 37 | hsa-miR-641 | 1.735 | 9.03E-05 | 0.000396304 |
| 38 | ggo-miR-106a | 1.907 | 9.05E-05 | 0.000396304 |
| 39 | hsa-miR-23b* | 1.340 | 9.21E-05 | 0.000401144 |
| 40 | hsa-miR-212 | 2.497 | 9.62E-05 | 0.000411712 |
| 41 | hsa-miR-373* | 2.556 | 0.00010218 | 0.000430272 |
| 42 | mml-miR-762 | 2.301 | 0.00011131 | 0.000452728 |
| 43 | hsa-miR-181d | 3.483 | 0.000121259 | 0.000484411 |
| 44 | hsa-miR-4253 | 2.876 | 0.000123722 | 0.000491715 |
| 45 | hsa-miR-92a-1* | 3.778 | 0.000144826 | 0.000553478 |
| 46 | hsa-miR-455-5p | 3.132 | 0.000150664 | 0.000569583 |
| 47 | hsa-miR-671-3p | 1.633 | 0.000153075 | 0.000573107 |
| 48 | hsa-miR-3195 | 2.883 | 0.000156033 | 0.000581373 |
| 49 | hsa-miR-92b | 2.413 | 0.00016166 | 0.000599458 |
| 50 | hsa-miR-365 | 3.755 | 0.000164678 | 0.000605925 |
| 51 | hsa-miR-1469 | 2.499 | 0.000164968 | 0.000605925 |
| 52 | hsa-miR-29b-1* | 4.006 | 0.000184132 | 0.000654598 |
| 53 | hsa-miR-1274a | 4.163 | 0.000188123 | 0.000664621 |
| 54 | hsa-miR-25 | 2.141 | 0.00019008 | 0.000664621 |
| 55 | ggo-miR-17-5p | 1.792 | 0.00019582 | 0.000675015 |
| 56 | hsa-miR-4281 | 1.414 | 0.000206177 | 0.000703907 |
| 57 | hsa-miR-4321 | 1.294 | 0.000233742 | 0.00078082 |
| 58 | hsa-miR-34a* | 2.271 | 0.000270089 | 0.000868544 |
| 59 | hsa-miR-1538 | 1.631 | 0.000343519 | 0.001027904 |
| 60 | hsa-miR-93* | 1.745 | 0.000385729 | 0.001115449 |
| 61 | hsa-miR-3124 | 1.574 | 0.000397854 | 0.001146233 |
| 62 | hsa-miR-24-2* | 2.504 | 0.000496985 | 0.001351188 |
| 63 | ppy-miR-1268 | 1.591 | 0.000498632 | 0.001351188 |
| 64 | ppy-miR-1181 | 1.478 | 0.000549452 | 0.001461012 |
| 65 | hsa-miR-28-3p | 1.683 | 0.000690034 | 0.001735446 |
| 66 | hsa-miR-4258 | 1.424 | 0.000700537 | 0.001735446 |
| 67 | mne-miR-197 | 2.649 | 0.000750523 | 0.001817673 |
| 68 | hsa-miR-1307 | 2.598 | 0.000781142 | 0.001877965 |
| 69 | hsa-miR-132 | 3.207 | 0.000810169 | 0.001908453 |
| 70 | hsa-miR-106b | 1.856 | 0.000836124 | 0.001957692 |
| 71 | hsa-miR-34c-3p | 4.626 | 0.000899894 | 0.002094349 |
| 72 | hsa-miR-342-3p | 1.145 | 0.000935735 | 0.002171241 |
| 73 | hsa-miR-1226 | 2.433 | 0.000951721 | 0.002192408 |
| 74 | hsa-miR-182 | 2.365 | 0.000953656 | 0.002192408 |
| 75 | hsa-miR-3185 | 1.896 | 0.001030981 | 0.00235003 |
| 76 | hsa-miR-183 | 2.639 | 0.001151598 | 0.002557274 |
| 77 | ppy-miR-298 | 1.712 | 0.001174597 | 0.002593484 |
| 78 | hsa-miR-933 | 1.757 | 0.001182798 | 0.002604171 |
| 79 | hsa-miR-19a | 2.539 | 0.001258724 | 0.002747816 |
| 80 | hsa-miR-184 | 2.311 | 0.001298057 | 0.002817911 |
| 81 | ppy-miR-331-3p | 1.784 | 0.001363332 | 0.002926821 |
| 82 | hsa-miR-34b* | 3.234 | 0.001753667 | 0.003643678 |
| 83 | ggo-miR-183 | 2.941 | 0.001762088 | 0.003651386 |
| 84 | hsa-miR-455-3p | 2.265 | 0.001925522 | 0.003916745 |
| 85 | hsa-miR-365* | 0.722 | 0.00198548 | 0.004028133 |
| 86 | ggo-miR-181b | 1.981 | 0.002014936 | 0.00407722 |
| 87 | hsa-miR-1260 | 5.124 | 0.00209973 | 0.004216196 |
| 88 | mml-miR-1235 | 1.313 | 0.002222326 | 0.004416161 |
| 89 | ptr-miR-1236 | 0.920 | 0.00226602 | 0.004480015 |
| 90 | ptr-miR-25 | 2.472 | 0.002359484 | 0.004627404 |
| 91 | hsa-miR-20b | 2.237 | 0.002360005 | 0.004627404 |
| 92 | hsa-miR-1228* | 1.556 | 0.002721758 | 0.005221195 |
| 93 | hsa-miR-130b | 3.093 | 0.002828305 | 0.005373611 |
| 94 | ggo-miR-20 | 1.642 | 0.002865493 | 0.005403302 |
| 95 | hsa-miR-191 | 1.034 | 0.002960539 | 0.005568974 |
| 96 | hsa-miR-330-3p | 1.713 | 0.003024714 | 0.005662206 |
| 97 | ppy-miR-182 | 1.931 | 0.003306215 | 0.006057486 |
| 98 | ppy-miR-1238 | 1.288 | 0.003474072 | 0.006290667 |
| 99 | hsa-miR-181b | 2.167 | 0.004058657 | 0.007181414 |
| 100 | hsa-miR-3180-5p | 1.416 | 0.00420535 | 0.007373633 |
| 101 | age-miR-222 | 1.017 | 0.00425144 | 0.007437621 |
| 102 | hsa-miR-19b | 1.444 | 0.00439972 | 0.007679691 |
| 103 | hsa-miR-34a | 1.648 | 0.004969791 | 0.008483675 |
| 104 | hsa-miR-1238 | 1.333 | 0.005203132 | 0.008759201 |
| 105 | hsa-miR-222 | 0.825 | 0.005837008 | 0.009530917 |
| 106 | hsa-miR-301a | 1.587 | 0.00584153 | 0.009530917 |
| 107 | hsa-miR-425 | 1.635 | 0.006428618 | 0.01031507 |
| 108 | hsa-miR-193a-5p | 1.279 | 0.007565467 | 0.011868901 |
| 109 | hsa-miR-602 | 2.293 | 0.007635046 | 0.01192976 |
| 110 | hsa-miR-4312 | 1.157 | 0.008566434 | 0.013277973 |
| 111 | hsa-miR-637 | 1.621 | 0.012761068 | 0.018555023 |
| 112 | hsa-miR-2355 | 1.955 | 0.012883229 | 0.01869757 |
| 113 | hsa-miR-550 | 1.246 | 0.013959125 | 0.019960004 |
| 114 | hsa-miR-1275 | 1.079 | 0.015546587 | 0.021946458 |
| 115 | hsa-miR-563 | 0.848 | 0.015699438 | 0.022081787 |
| 116 | hsa-miR-2861 | 0.809 | 0.019426516 | 0.026980785 |
| 117 | hsa-miR-331-3p | 1.170 | 0.02270814 | 0.031093301 |
| 118 | ppy-miR-720 | 1.097 | 0.024272047 | 0.033001467 |
| 119 | ppy-miR-602 | 2.793 | 0.025962138 | 0.035053409 |
| 120 | hsa-miR-4310 | 1.184 | 0.028171237 | 0.037772853 |
| 121 | hsa-miR-296-5p | 1.092 | 0.028848284 | 0.038547275 |
| 122 | ptr-miR-1225 | 1.177 | 0.029555247 | 0.039356214 |
| 123 | ppy-miR-1909 | 1.634 | 0.0296438 | 0.039406423 |
| 124 | hsa-miR-4290 | 1.563 | 0.03093214 | 0.040908547 |
| 125 | hsa-miR-106b* | 1.255 | 0.032228198 | 0.042477642 |
| 126 | hsa-miR-320d | 1.020 | 0.032450362 | 0.042697845 |

|  | **Table 2b: AGMK1-9T7 miRNAs whose expression was significantly decreased at p2 (q<0.05)** | | | |
| --- | --- | --- | --- | --- |
|  |  |  |  |  |
|  | **miRNA** | **Log2(p2v0 fold change)** | **p2v0 pval** | **p2v0 qval** |
| 1 | hsa-miR-1973 | -10.191 | 9.44E-13 | 3.66E-10 |
| 2 | hsa-miR-199a-5p | -10.385 | 1.69E-11 | 3.28E-09 |
| 3 | hsa-miR-199a-3p | -6.161 | 6.30E-10 | 8.13E-08 |
| 4 | hsa-miR-145 | -6.105 | 4.64E-09 | 3.60E-07 |
| 5 | mml-miR-512-5p | -3.878 | 2.77E-08 | 1.69E-06 |
| 6 | ggo-miR-143 | -5.055 | 2.83E-08 | 1.69E-06 |
| 7 | ggo-miR-145 | -6.028 | 4.36E-08 | 2.42E-06 |
| 8 | hsa-miR-152 | -2.970 | 8.75E-08 | 4.24E-06 |
| 9 | hsa-miR-1207-5p | -5.409 | 1.30E-07 | 4.59E-06 |
| 10 | hsa-miR-568 | -6.320 | 1.36E-07 | 4.59E-06 |
| 11 | hsa-miR-142-3p | -5.470 | 1.93E-07 | 5.54E-06 |
| 12 | ppa-miR-141 | -2.703 | 2.22E-07 | 6.13E-06 |
| 13 | hsa-miR-192* | -6.170 | 2.54E-07 | 6.78E-06 |
| 14 | hsa-miR-499-5p | -7.623 | 3.08E-07 | 7.71E-06 |
| 15 | ptr-miR-602 | -5.015 | 4.82E-07 | 1.10E-05 |
| 16 | hsa-miR-542-5p | -3.719 | 4.99E-07 | 1.11E-05 |
| 17 | hsa-miR-1285 | -8.201 | 5.64E-07 | 1.18E-05 |
| 18 | hsa-miR-126 | -7.071 | 6.04E-07 | 1.23E-05 |
| 19 | hsa-miR-142-5p | -7.206 | 1.37E-06 | 2.46E-05 |
| 20 | ppa-miR-188 | -1.477 | 1.93E-06 | 3.06E-05 |
| 21 | hsa-miR-1273c | -5.620 | 2.09E-06 | 3.11E-05 |
| 22 | hsa-miR-219-2-3p | -4.653 | 3.30E-06 | 4.13E-05 |
| 23 | hsa-miR-194* | -5.109 | 3.54E-06 | 4.28E-05 |
| 24 | hsa-miR-148a | -4.952 | 5.00E-06 | 5.79E-05 |
| 25 | ggo-miR-141 | -2.100 | 5.58E-06 | 6.09E-05 |
| 26 | ptr-miR-631 | -3.369 | 6.11E-06 | 6.52E-05 |
| 27 | ptr-miR-661 | -4.059 | 7.63E-06 | 7.58E-05 |
| 28 | mml-miR-604 | -4.624 | 8.63E-06 | 8.26E-05 |
| 29 | hsa-miR-187 | -6.624 | 1.15E-05 | 0.000102071 |
| 30 | hsa-miR-4287 | -5.605 | 1.20E-05 | 0.000104537 |
| 31 | hsa-miR-99a | -2.928 | 1.22E-05 | 0.000104537 |
| 32 | hsa-miR-126* | -9.465 | 1.25E-05 | 0.000104537 |
| 33 | hsa-miR-214 | -5.227 | 1.28E-05 | 0.000104537 |
| 34 | ppy-miR-520c-5p | -5.316 | 1.29E-05 | 0.000104537 |
| 35 | ppy-miR-199b-3p | -8.152 | 1.43E-05 | 0.000111565 |
| 36 | hsa-miR-4285 | -4.663 | 1.74E-05 | 0.000128352 |
| 37 | pbi-miR-513c | -5.080 | 1.97E-05 | 0.000142407 |
| 38 | hsa-miR-362-3p | -3.170 | 3.33E-05 | 0.000199837 |
| 39 | hsa-miR-653 | -5.177 | 3.57E-05 | 0.000208081 |
| 40 | hsa-miR-1975 | -1.553 | 4.31E-05 | 0.000240161 |
| 41 | hsa-miR-139-3p | -3.524 | 4.51E-05 | 0.000244508 |
| 42 | hsa-miR-500* | -3.280 | 4.55E-05 | 0.000244508 |
| 43 | hsa-miR-143 | -5.891 | 5.03E-05 | 0.000259934 |
| 44 | age-miR-198 | -3.780 | 5.84E-05 | 0.000287463 |
| 45 | hsa-miR-150 | -5.999 | 6.30E-05 | 0.000304116 |
| 46 | hsa-miR-451 | -12.240 | 7.08E-05 | 0.00033145 |
| 47 | hsa-miR-101 | -3.468 | 7.66E-05 | 0.000349164 |
| 48 | hsa-miR-513a-5p | -8.834 | 8.22E-05 | 0.00037244 |
| 49 | hsa-miR-500 | -2.149 | 8.34E-05 | 0.000374143 |
| 50 | ggo-miR-101 | -2.825 | 9.40E-05 | 0.000406797 |
| 51 | hsa-miR-26b* | -4.833 | 0.000102034 | 0.000430272 |
| 52 | hsa-miR-26a | -1.532 | 0.000109918 | 0.000452728 |
| 53 | hsa-miR-3130-3p | -6.531 | 0.000111229 | 0.000452728 |
| 54 | hsa-miR-196a | -3.029 | 0.000111576 | 0.000452728 |
| 55 | ppy-miR-513c | -7.292 | 0.000114771 | 0.000460866 |
| 56 | hsa-miR-422a | -2.994 | 0.000134052 | 0.000522063 |
| 57 | ggo-miR-30b | -1.373 | 0.000137857 | 0.00053154 |
| 58 | hsa-miR-200a | -2.800 | 0.000144976 | 0.000553478 |
| 59 | hsa-miR-765 | -3.244 | 0.000147872 | 0.000561768 |
| 60 | hsa-miR-1246 | -2.346 | 0.000151971 | 0.000571737 |
| 61 | mml-miR-765 | -3.418 | 0.000189632 | 0.000664621 |
| 62 | hsa-miR-572 | -3.149 | 0.000208066 | 0.000707243 |
| 63 | hsa-miR-483-5p | -3.827 | 0.000223046 | 0.000754851 |
| 64 | hsa-miR-2115* | -5.066 | 0.000231892 | 0.000777991 |
| 65 | hsa-miR-141 | -2.061 | 0.000248906 | 0.00082437 |
| 66 | hsa-miR-223 | -3.561 | 0.000251748 | 0.000830232 |
| 67 | hsa-miR-4275 | -4.939 | 0.000264242 | 0.000860453 |
| 68 | mml-miR-297 | -3.369 | 0.000267378 | 0.000863408 |
| 69 | hsa-miR-660 | -3.421 | 0.00027623 | 0.00087766 |
| 70 | ggo-miR-214 | -4.986 | 0.000317036 | 0.000971158 |
| 71 | ppy-miR-1254 | -3.199 | 0.00034182 | 0.001026784 |
| 72 | hsa-miR-138 | -3.131 | 0.000355234 | 0.001050789 |
| 73 | hsa-miR-10b | -3.035 | 0.000373153 | 0.00109543 |
| 74 | hsa-miR-608 | -3.170 | 0.000378565 | 0.001102961 |
| 75 | hsa-miR-3193 | -3.773 | 0.000402089 | 0.001149886 |
| 76 | hsa-miR-218 | -2.872 | 0.000406465 | 0.001158125 |
| 77 | hsa-miR-1303 | -1.621 | 0.00042466 | 0.001192434 |
| 78 | hsa-miR-30a | -1.363 | 0.000517936 | 0.00139375 |
| 79 | ggo-miR-10b | -3.841 | 0.000525912 | 0.001410318 |
| 80 | hsa-miR-204 | -6.604 | 0.00055122 | 0.001461012 |
| 81 | ppy-miR-10a | -2.323 | 0.000552357 | 0.001461012 |
| 82 | hsa-miR-30c | -1.389 | 0.000562859 | 0.001478696 |
| 83 | hsa-let-7f | -1.436 | 0.00058285 | 0.001526042 |
| 84 | mml-miR-936 | -2.266 | 0.000588838 | 0.001536529 |
| 85 | hsa-miR-1471 | -3.053 | 0.000619221 | 0.001599653 |
| 86 | ggo-miR-200c | -1.113 | 0.000650342 | 0.001668923 |
| 87 | hsa-miR-486-5p | -2.261 | 0.000652584 | 0.001669151 |
| 88 | hsa-miR-200c | -1.349 | 0.000695822 | 0.001735446 |
| 89 | hsa-miR-98 | -2.491 | 0.00070013 | 0.001735446 |
| 90 | ppy-miR-510 | -4.369 | 0.000702408 | 0.001735446 |
| 91 | ppy-miR-1246 | -2.289 | 0.000715511 | 0.001760385 |
| 92 | hsa-miR-26b | -3.620 | 0.000725335 | 0.001778908 |
| 93 | hsa-miR-215 | -4.877 | 0.000782687 | 0.001877965 |
| 94 | hsa-miR-29c* | -2.802 | 0.000890682 | 0.002079152 |
| 95 | hsa-miR-711 | -4.611 | 0.000943619 | 0.002182999 |
| 96 | hsa-miR-1323 | -2.226 | 0.000956173 | 0.002192408 |
| 97 | hsa-miR-623 | -1.743 | 0.00104051 | 0.002364796 |
| 98 | hsa-miR-30d | -1.643 | 0.001105094 | 0.002475282 |
| 99 | hsa-miR-452 | -3.301 | 0.001144677 | 0.002549209 |
| 100 | hsa-miR-30b | -1.506 | 0.001258877 | 0.002747816 |
| 101 | hsa-miR-10a | -2.039 | 0.001262223 | 0.002747816 |
| 102 | mml-miR-211 | -5.261 | 0.001304844 | 0.002824732 |
| 103 | hsa-miR-3138 | -3.874 | 0.001337071 | 0.002886435 |
| 104 | ggo-miR-223 | -1.868 | 0.001367806 | 0.002928314 |
| 105 | hsa-miR-532-5p | -2.695 | 0.001586906 | 0.003314965 |
| 106 | hsa-miR-30e | -1.951 | 0.001830565 | 0.003773105 |
| 107 | hsa-miR-3173 | -4.728 | 0.001878801 | 0.003836379 |
| 108 | hsa-miR-424 | -2.876 | 0.001881063 | 0.003836379 |
| 109 | hsa-miR-4315 | -2.263 | 0.002364454 | 0.004627404 |
| 110 | hsa-miR-371-5p | -1.326 | 0.002845 | 0.005381454 |
| 111 | hsa-miR-3194 | -2.683 | 0.002846963 | 0.005381454 |
| 112 | hsa-miR-196b* | -2.531 | 0.003016514 | 0.005660529 |
| 113 | hsa-miR-4288 | -1.874 | 0.003079007 | 0.005736131 |
| 114 | hsa-let-7a | -1.172 | 0.003090667 | 0.005741292 |
| 115 | hsa-miR-532-3p | -3.533 | 0.003420628 | 0.006231145 |
| 116 | hsa-miR-194 | -2.149 | 0.003610111 | 0.006506596 |
| 117 | hsa-miR-886-3p | -2.501 | 0.003715048 | 0.006649335 |
| 118 | hsa-miR-192 | -2.617 | 0.00388776 | 0.006910583 |
| 119 | hsa-miR-200b | -1.649 | 0.004004852 | 0.007102426 |
| 120 | hsa-miR-381 | -2.516 | 0.004641064 | 0.008064629 |
| 121 | hsa-miR-30a* | -1.731 | 0.00466951 | 0.008077836 |
| 122 | hsa-miR-362-5p | -1.952 | 0.004763359 | 0.008203563 |
| 123 | ggo-miR-29b | -1.982 | 0.004775168 | 0.008205666 |
| 124 | hsa-let-7g | -1.120 | 0.005200363 | 0.008759201 |
| 125 | ptr-miR-670 | -4.044 | 0.005201718 | 0.008759201 |
| 126 | hsa-let-7d | -1.199 | 0.005210584 | 0.008759201 |
| 127 | hsa-miR-188-5p | -1.565 | 0.005242087 | 0.008774552 |
| 128 | hsa-miR-574-5p | -2.560 | 0.005253753 | 0.008775126 |
| 129 | hsa-miR-195 | -2.868 | 0.005351397 | 0.00887027 |
| 130 | hsa-miR-4306 | -1.680 | 0.005367944 | 0.00887027 |
| 131 | hsa-let-7e | -1.535 | 0.005586433 | 0.009211672 |
| 132 | hsa-let-7c | -1.542 | 0.005806285 | 0.00951347 |
| 133 | hsa-miR-30e* | -2.818 | 0.005967999 | 0.009716806 |
| 134 | hsa-miR-2114* | -4.000 | 0.006375348 | 0.01029353 |
| 135 | hsa-miR-4330 | -4.470 | 0.006414 | 0.010312967 |
| 136 | hsa-miR-1321 | -4.728 | 0.006617963 | 0.010575095 |
| 137 | hsa-miR-187* | -1.613 | 0.007082133 | 0.011201333 |
| 138 | hsa-miR-196a* | -3.874 | 0.007433854 | 0.011709832 |
| 139 | hsa-miR-361-5p | -1.452 | 0.007626911 | 0.01192976 |
| 140 | hsa-miR-125b | -1.433 | 0.008173703 | 0.01272012 |
| 141 | hsa-miR-4328 | -6.556 | 0.008492273 | 0.013189403 |
| 142 | hsa-miR-3172 | -8.293 | 0.008628694 | 0.01334778 |
| 143 | hsa-miR-148b | -1.978 | 0.008760912 | 0.013525313 |
| 144 | hsa-miR-3177 | -1.952 | 0.008960434 | 0.013778446 |
| 145 | ggo-miR-186 | -2.499 | 0.009170333 | 0.014045471 |
| 146 | hsa-miR-186 | -2.263 | 0.009265112 | 0.014162647 |
| 147 | hsa-miR-502-3p | -3.348 | 0.009323793 | 0.014224291 |
| 148 | hsa-miR-4324 | -2.016 | 0.009382223 | 0.014285311 |
| 149 | ppy-miR-1303 | -6.887 | 0.010081702 | 0.01520101 |
| 150 | hsa-miR-125a-5p | -0.972 | 0.010273799 | 0.015410053 |
| 151 | hsa-miR-29c | -1.119 | 0.010279996 | 0.015410053 |
| 152 | hsa-miR-514b-5p | -7.072 | 0.010368213 | 0.015482398 |
| 153 | hsa-miR-376a | -2.981 | 0.010624383 | 0.015804025 |
| 154 | hsa-miR-429 | -1.422 | 0.011867926 | 0.017486013 |
| 155 | hsa-let-7b | -1.353 | 0.012479679 | 0.018214221 |
| 156 | hsa-miR-489 | -2.038 | 0.013158133 | 0.018962931 |
| 157 | ssy-miR-513c | -3.807 | 0.013668717 | 0.019580879 |
| 158 | hsa-miR-10b* | -3.063 | 0.014494042 | 0.020648681 |
| 159 | hsa-miR-518c* | -4.010 | 0.014881167 | 0.021083921 |
| 160 | hsa-miR-1308 | -1.252 | 0.015851683 | 0.022255533 |
| 161 | hsa-miR-29b | -1.800 | 0.016077413 | 0.022490966 |
| 162 | hsa-miR-378 | -1.635 | 0.018738763 | 0.026072786 |
| 163 | hsa-miR-377 | -1.800 | 0.019460979 | 0.026980785 |
| 164 | hsa-miR-4300 | -4.129 | 0.02010341 | 0.027722674 |
| 165 | hsa-miR-135a* | -4.694 | 0.023931553 | 0.0325957 |
| 166 | hsa-miR-136* | -4.196 | 0.025777744 | 0.034865186 |
| 167 | hsa-miR-875-5p | -6.055 | 0.029247084 | 0.039012891 |
| 168 | hsa-miR-15a | -1.056 | 0.030354194 | 0.040281678 |
| 169 | ppy-miR-647 | -6.235 | 0.03081983 | 0.04082969 |
| 170 | ppy-miR-564 | -1.080 | 0.0334743 | 0.04382193 |

|  | **Table 3a: AGMK1-9T7 miRNAs whose expression was significantly increased at p10 (q<0.05)** | | | |
| --- | --- | --- | --- | --- |
|  |  |  |  |  |
|  |  |  |  |  |
|  | **miRNA** | **Log2(p10v0 fold change)** | **p10v0 pval** | **p10v0 qval** |
| 1 | hsa-miR-34c-3p | 7.123 | 6.51E-10 | 1.64E-08 |
| 2 | hsa-miR-34c-5p | 7.914 | 1.46E-09 | 2.90E-08 |
| 3 | hsa-miR-3195 | 4.885 | 1.64E-08 | 2.34E-07 |
| 4 | hsa-miR-7-1* | 5.994 | 3.33E-08 | 4.10E-07 |
| 5 | hsa-miR-15b | 1.498 | 2.23E-07 | 1.95E-06 |
| 6 | hsa-miR-466 | 5.143 | 3.61E-07 | 2.95E-06 |
| 7 | ggo-miR-93 | 2.518 | 1.77E-06 | 1.17E-05 |
| 8 | hsa-miR-181c | 3.594 | 1.78E-06 | 1.17E-05 |
| 9 | hsa-miR-130b | 3.304 | 2.02E-06 | 1.29E-05 |
| 10 | hsa-miR-181a | 1.924 | 2.11E-06 | 1.33E-05 |
| 11 | hsa-miR-31 | 2.574 | 2.35E-05 | 0.000113205 |
| 12 | ggo-miR-31 | 2.631 | 3.02E-05 | 0.000138609 |
| 13 | hsa-miR-93 | 2.486 | 3.14E-05 | 0.000142678 |
| 14 | hsa-miR-34a | 2.203 | 0.000464838 | 0.00160239 |
| 15 | hsa-miR-484 | 1.537 | 0.000691659 | 0.002298627 |
| 16 | age-miR-222 | 2.436 | 0.000743461 | 0.002441543 |
| 17 | hsa-miR-3178 | 1.275 | 0.001383696 | 0.004135387 |
| 18 | hsa-miR-221 | 1.562 | 0.002890908 | 0.008103304 |
| 19 | hsa-miR-106b | 2.145 | 0.004231189 | 0.011182428 |
| 20 | hsa-miR-92a | 0.843 | 0.005934662 | 0.014704184 |
| 21 | hsa-miR-222 | 2.246 | 0.009247171 | 0.021600495 |
| 22 | hsa-miR-425 | 2.979 | 0.015138401 | 0.033208746 |
| 23 | hsa-miR-18a | 3.534 | 0.01774829 | 0.037885773 |

|  | **Table 3b: AGMK1-9T7 miRNAs whose expression was significantly decreased at p10 (q<0.05)** | | | |
| --- | --- | --- | --- | --- |
|  |  |  |  |  |
|  |  |  |  |  |
|  | **miRNA** | **Log2(p10v0 fold change)** | **p10v0 pval** | **p10v0 qval** |
| 1 | ggo-miR-10b | -7.939 | 1.06E-12 | 1.96E-10 |
| 2 | ppa-miR-141 | -9.626 | 2.18E-11 | 1.51E-09 |
| 3 | ggo-miR-141 | -9.364 | 6.73E-11 | 3.40E-09 |
| 4 | hsa-miR-1207-5p | -6.376 | 9.56E-11 | 4.42E-09 |
| 5 | hsa-miR-148a | -6.622 | 1.87E-10 | 6.48E-09 |
| 6 | hsa-miR-100 | -3.808 | 5.21E-10 | 1.56E-08 |
| 7 | hsa-miR-125b | -4.919 | 5.36E-10 | 1.56E-08 |
| 8 | hsa-miR-10b | -4.775 | 5.61E-10 | 1.56E-08 |
| 9 | hsa-miR-126 | -5.429 | 2.32E-09 | 4.44E-08 |
| 10 | hsa-let-7c | -3.868 | 8.17E-09 | 1.30E-07 |
| 11 | hsa-miR-514b-5p | -6.835 | 8.93E-09 | 1.34E-07 |
| 12 | hsa-miR-3196 | -2.867 | 4.02E-08 | 4.64E-07 |
| 13 | ppy-miR-1274b | -3.001 | 7.28E-07 | 5.54E-06 |
| 14 | ppy-miR-10a | -3.000 | 1.11E-06 | 7.81E-06 |
| 15 | hsa-miR-557 | -4.131 | 3.64E-06 | 2.20E-05 |
| 16 | hsa-miR-195 | -3.873 | 5.82E-06 | 3.34E-05 |
| 17 | hsa-miR-495 | -2.169 | 6.23E-06 | 3.53E-05 |
| 18 | hsa-miR-4257 | -4.705 | 9.09E-06 | 4.85E-05 |
| 19 | hsa-miR-200a | -6.465 | 1.39E-05 | 7.15E-05 |
| 20 | hsa-miR-1275 | -2.501 | 1.54E-05 | 7.77E-05 |
| 21 | hsa-miR-1308 | -4.294 | 2.24E-05 | 0.00011011 |
| 22 | ppy-miR-1268 | -3.347 | 2.46E-05 | 0.00011766 |
| 23 | hsa-miR-638 | -3.246 | 3.00E-05 | 0.000138609 |
| 24 | hsa-miR-373* | -3.093 | 5.24E-05 | 0.000223811 |
| 25 | mml-miR-512-5p | -5.415 | 7.26E-05 | 0.000292072 |
| 26 | hsa-miR-10a | -1.783 | 0.000135091 | 0.000520185 |
| 27 | mml-miR-765 | -1.648 | 0.000174508 | 0.000654405 |
| 28 | hsa-miR-487b | -1.900 | 0.000229013 | 0.000849889 |
| 29 | hsa-miR-711 | -4.289 | 0.000483988 | 0.001658108 |
| 30 | hsa-let-7d | -2.580 | 0.001278012 | 0.003897234 |
| 31 | hsa-miR-204 | -4.777 | 0.001313779 | 0.003984413 |
| 32 | hsa-miR-3172 | -6.931 | 0.001860844 | 0.005362789 |
| 33 | hsa-miR-98 | -2.572 | 0.002933485 | 0.008181327 |
| 34 | hsa-let-7b | -4.291 | 0.004161145 | 0.011103056 |
| 35 | ppy-miR-1303 | -6.302 | 0.004546667 | 0.011772984 |
| 36 | hsa-miR-193a-5p | -3.508 | 0.004560705 | 0.011772984 |
| 37 | hsa-miR-762 | -1.138 | 0.005297345 | 0.013501962 |
| 38 | hsa-miR-4328 | -6.649 | 0.005427498 | 0.013568745 |
| 39 | hsa-miR-939 | -4.533 | 0.006189259 | 0.01515643 |
| 40 | hsa-let-7a | -2.478 | 0.006199116 | 0.01515643 |
| 41 | hsa-miR-1228* | -4.488 | 0.007345868 | 0.017649163 |
| 42 | hsa-let-7e | -2.466 | 0.007647616 | 0.018294944 |
| 43 | hsa-miR-429 | -4.192 | 0.008368168 | 0.019679377 |
| 44 | ggo-miR-200c | -9.638 | 0.01078926 | 0.024541144 |
| 45 | ppy-miR-1207-5p | -3.597 | 0.012909027 | 0.029124023 |
| 46 | hsa-miR-3185 | -3.466 | 0.013393121 | 0.029889146 |
| 47 | hsa-miR-126* | -8.558 | 0.015207256 | 0.033228454 |
| 48 | mml-miR-1227 | -3.339 | 0.015367016 | 0.033445859 |
| 49 | hsa-miR-1975 | -1.522 | 0.019131487 | 0.040374839 |
| 50 | hsa-miR-200b | -5.468 | 0.01960176 | 0.041052743 |
| 51 | hsa-miR-1231 | -3.867 | 0.022529719 | 0.046140199 |
| 52 | hsa-miR-30d | -1.279 | 0.023066705 | 0.04689385 |

|  | **Table 4a: AGMK1-9T7 miRNAs whose expression was significantly increased at p20 (q<0.05)** | | | |
| --- | --- | --- | --- | --- |
|  |  |  |  |  |
|  | **miRNA** | **Log2(p20v0 fold change)** | **p20v0 pval** | **p20v0 qval** |
| 1 | hsa-miR-34c-3p | 5.802 | 3.82E-10 | 1.25E-08 |
| 2 | hsa-miR-181c | 2.996 | 8.57E-09 | 1.32E-07 |
| 3 | hsa-miR-487b | 2.200 | 1.25E-07 | 1.22E-06 |
| 4 | age-miR-222 | 2.368 | 1.58E-07 | 1.43E-06 |
| 5 | hsa-miR-7-1* | 5.291 | 2.28E-07 | 1.95E-06 |
| 6 | hsa-miR-485-3p | 4.004 | 2.33E-07 | 1.95E-06 |
| 7 | hsa-miR-3195 | 5.033 | 8.02E-07 | 6.01E-06 |
| 8 | hsa-miR-155 | 2.968 | 1.24E-06 | 8.61E-06 |
| 9 | ggo-miR-18 | 3.298 | 1.79E-06 | 1.17E-05 |
| 10 | hsa-miR-34c-5p | 6.968 | 3.42E-06 | 2.09E-05 |
| 11 | hsa-miR-18a | 4.026 | 4.10E-06 | 2.42E-05 |
| 12 | hsa-miR-425 | 2.978 | 5.84E-06 | 3.34E-05 |
| 13 | hsa-miR-130b | 4.247 | 7.06E-06 | 3.92E-05 |
| 14 | hsa-miR-484 | 2.624 | 2.55E-05 | 0.000119873 |
| 15 | hsa-miR-654-3p | 2.348 | 3.65E-05 | 0.000162461 |
| 16 | hsa-miR-3178 | 1.451 | 4.24E-05 | 0.000185746 |
| 17 | hsa-miR-17 | 0.991 | 0.000239943 | 0.000876109 |
| 18 | hsa-miR-331-3p | 1.245 | 0.000332328 | 0.001167355 |
| 19 | hsa-miR-197 | 1.759 | 0.000708204 | 0.002339601 |
| 20 | ggo-miR-181b | 1.326 | 0.001747414 | 0.005104289 |
| 21 | hsa-miR-466 | 3.971 | 0.002160286 | 0.006148507 |
| 22 | hsa-miR-222 | 2.280 | 0.00415249 | 0.011103056 |
| 23 | ggo-miR-183 | 0.533 | 0.00586559 | 0.014598217 |
| 24 | hsa-miR-221 | 1.573 | 0.006529789 | 0.015894881 |
| 25 | hsa-miR-181a | 1.571 | 0.006953817 | 0.016779863 |
| 26 | ggo-miR-93 | 2.500 | 0.010574974 | 0.024252523 |
| 27 | hsa-miR-543 | 2.644 | 0.019370134 | 0.040721304 |
| 28 | hsa-miR-379 | 2.005 | 0.020427186 | 0.04237558 |
| 29 | hsa-miR-93 | 2.449 | 0.02202709 | 0.045277908 |
| 30 | hsa-miR-495 | 2.282 | 0.02339468 | 0.047214717 |
| 31 | hsa-miR-106a | 1.012 | 0.023894936 | 0.047876135 |
| 32 | hsa-miR-4298 | 2.216 | 0.024528757 | 0.048969282 |

|  | **Table 4b: AGMK1-9T7 miRNAs whose expression was significantly decreased at p20 (q<0.05)** | | | | | | | |
| --- | --- | --- | --- | --- | --- | --- | --- | --- |
|  |  | |  | |  | |  | |
|  | **miRNA** | | **Log2(p20v0 fold change)** | | **p20v0 pval** | | **p20v0 qval** | |
| 1 | ggo-miR-29b | | -11.582 | | 4.70E-14 | | 2.61E-11 | |
| 2 | hsa-miR-99a | | -10.741 | | 3.11E-12 | | 3.45E-10 | |
| 3 | hsa-miR-3172 | | -11.516 | | 2.05E-11 | | 1.51E-09 | |
| 4 | ppa-miR-141 | | -9.626 | | 2.18E-11 | | 1.51E-09 | |
| 5 | hsa-miR-126 | | -8.552 | | 5.05E-11 | | 3.12E-09 | |
| 6 | hsa-miR-514b-5p | | -9.295 | | 6.35E-10 | | 1.64E-08 | |
| 7 | hsa-miR-196a | | -4.021 | | 1.38E-09 | | 2.90E-08 | |
| 8 | hsa-let-7b | | -3.939 | | 3.96E-09 | | 7.32E-08 | |
| 9 | hsa-let-7d | | -2.697 | | 5.53E-09 | | 9.90E-08 | |
| 10 | hsa-let-7a | | -2.526 | | 5.81E-09 | | 1.01E-07 | |
| 11 | hsa-miR-29b | | -5.209 | | 7.97E-09 | | 1.30E-07 | |
| 12 | hsa-miR-1469 | | -3.260 | | 1.73E-08 | | 2.40E-07 | |
| 13 | hsa-miR-145 | | -6.802 | | 3.42E-08 | | 4.10E-07 | |
| 14 | mml-miR-512-5p | | -6.628 | | 3.47E-08 | | 4.10E-07 | |
| 15 | hsa-miR-150* | | -3.874 | | 4.31E-08 | | 4.88E-07 | |
| 16 | hsa-let-7f | | -1.851 | | 6.36E-08 | | 6.79E-07 | |
| 17 | hsa-miR-939 | | -7.855 | | 7.92E-08 | | 8.00E-07 | |
| 18 | ppy-miR-1207-5p | | -3.505 | | 3.95E-07 | | 3.13E-06 | |
| 19 | hsa-miR-1246 | | -2.061 | | 2.02E-06 | | 1.29E-05 | |
| 20 | ggo-miR-224 | | -3.103 | | 3.81E-06 | | 2.28E-05 | |
| 21 | hsa-miR-1228* | | -5.133 | | 9.29E-06 | | 4.90E-05 | |
| 22 | hsa-miR-1975 | | -1.700 | | 9.36E-06 | | 4.90E-05 | |
| 23 | hsa-miR-1268 | | -3.319 | | 1.65E-05 | | 8.25E-05 | |
| 24 | hsa-miR-187* | | -3.332 | | 2.50E-05 | | 0.000118584 | |
| 25 | hsa-miR-30e | | -2.613 | | 3.66E-05 | | 0.000162461 | |
| 26 | hsa-miR-100 | | -3.701 | | 5.19E-05 | | 0.000223403 | |
| 27 | hsa-miR-1915 | | -7.494 | | 6.76E-05 | | 0.000277789 | |
| 28 | hsa-miR-193a-5p | | -2.278 | | 0.000144319 | | 0.00054861 | |
| 29 | hsa-miR-30d | | -0.734 | | 0.000580949 | | 0.001966016 | |
| 30 | ppy-miR-298 | | -3.391 | | 0.000856093 | | 0.002770108 | |
| 31 | hsa-miR-1973 | | -1.000 | | 0.00116452 | | 0.003630946 | |
| 32 | hsa-let-7e | | -2.858 | | 0.001271962 | | 0.003897234 | |
| 33 | hsa-miR-26a | | -0.957 | | 0.001495553 | | 0.004438673 | |
| 34 | hsa-miR-3141 | | -2.472 | | 0.002091733 | | 0.005984081 | |
| 35 | hsa-miR-3185 | | -3.588 | | 0.004194603 | | 0.011138777 | |
| 36 | hsa-miR-2861 | | -4.327 | | 0.004318014 | | 0.011357809 | |
| 37 | hsa-miR-1207-5p | | -6.344 | | 0.004341263 | | 0.0113651 | |
| 38 | ggo-miR-214 | | -4.794 | | 0.004861505 | | 0.012491367 | |
| 39 | hsa-miR-141 | | -5.654 | | 0.005303473 | | 0.013501962 | |
| 40 | mml-miR-638 | | -1.532 | | 0.008243382 | | 0.019468413 | |
| 41 | hsa-miR-200c | | -8.256 | | 0.009450455 | | 0.021945618 | |
| 42 | hsa-miR-371-5p | | -8.405 | | 0.009600695 | | 0.022201606 | |
| 43 | hsa-miR-663 | | -5.798 | | 0.010493839 | | 0.02416631 | |
| 44 | ggo-miR-200c | | -9.638 | | 0.01078926 | | 0.024541144 | |
| 45 | hsa-miR-765 | | -1.837 | | 0.013347832 | | 0.029889146 | |
| 46 | hsa-miR-1293 | | -7.060 | | 0.015797909 | | 0.034249374 | |
| 47 | ggo-miR-30b | | -0.777 | | 0.016718342 | | 0.035963876 | |
| 48 | ppy-miR-1246 | | -2.408 | | 0.020462442 | | 0.04237558 | |
| 49 | hsa-miR-513a-5p | | -8.571 | | 0.022806112 | | 0.04653453 | |
| 50 | hsa-miR-30a | | -0.722 | | 0.023184093 | | 0.046960481 | |
|  | **Table 5a: AGMK1-9T7 miRNAs whose expression was significantly increased at p40 (q<.05)** | | | | | | |  |
|  |  |  | |  | |  | |  |
|  | **miRNA** | **Log2(p40v0 fold change)** | | **p40v0 pval** | | **p40v0 qval** | |  |
| 1 | hsa-miR-15b | 3.036 | | 6.84E-10 | | 1.65E-08 | |  |
| 2 | hsa-miR-299-5p | 4.641 | | 7.22E-09 | | 1.22E-07 | |  |
| 3 | hsa-miR-466 | 4.180 | | 1.61E-08 | | 2.34E-07 | |  |
| 4 | hsa-miR-34c-5p | 6.664 | | 2.28E-08 | | 3.09E-07 | |  |
| 5 | hsa-miR-485-3p | 5.255 | | 2.76E-08 | | 3.65E-07 | |  |
| 6 | hsa-miR-379 | 3.577 | | 3.16E-08 | | 4.08E-07 | |  |
| 7 | hsa-miR-181b | 1.967 | | 5.48E-08 | | 6.08E-07 | |  |
| 8 | hsa-miR-487b | 3.934 | | 7.90E-08 | | 8.00E-07 | |  |
| 9 | hsa-miR-130b | 4.275 | | 1.11E-07 | | 1.10E-06 | |  |
| 10 | hsa-miR-601 | 4.063 | | 1.37E-07 | | 1.30E-06 | |  |
| 11 | hsa-miR-3195 | 3.728 | | 1.38E-07 | | 1.30E-06 | |  |
| 12 | ggo-miR-181b | 1.679 | | 1.55E-07 | | 1.43E-06 | |  |
| 13 | age-miR-222 | 2.129 | | 1.76E-07 | | 1.58E-06 | |  |
| 14 | hsa-miR-7-1* | 6.253 | | 2.36E-07 | | 1.95E-06 | |  |
| 15 | hsa-miR-155 | 4.174 | | 5.32E-07 | | 4.16E-06 | |  |
| 16 | ggo-miR-183 | 2.101 | | 9.46E-07 | | 6.91E-06 | |  |
| 17 | hsa-miR-93 | 2.346 | | 1.34E-06 | | 9.16E-06 | |  |
| 18 | hsa-miR-495 | 4.883 | | 1.69E-06 | | 1.14E-05 | |  |
| 19 | hsa-miR-183 | 1.940 | | 2.83E-06 | | 1.77E-05 | |  |
| 20 | ggo-miR-93 | 2.320 | | 3.09E-06 | | 1.91E-05 | |  |
| 21 | hsa-miR-132 | 2.310 | | 5.79E-06 | | 3.34E-05 | |  |
| 22 | hsa-miR-329 | 3.853 | | 8.09E-06 | | 4.44E-05 | |  |
| 23 | hsa-miR-425 | 2.514 | | 8.32E-06 | | 4.49E-05 | |  |
| 24 | hsa-miR-221 | 1.248 | | 2.31E-05 | | 0.000112417 | |  |
| 25 | hsa-miR-411* | 5.366 | | 5.48E-05 | | 0.000230588 | |  |
| 26 | hsa-miR-92a | 1.551 | | 6.43E-05 | | 0.000266143 | |  |
| 27 | hsa-miR-484 | 2.245 | | 7.03E-05 | | 0.000285717 | |  |
| 28 | hsa-miR-21 | 2.493 | | 9.25E-05 | | 0.00036403 | |  |
| 29 | hsa-miR-222 | 1.932 | | 0.000126831 | | 0.000492248 | |  |
| 30 | hsa-miR-92b | 1.647 | | 0.000135904 | | 0.000520185 | |  |
| 31 | hsa-miR-654-3p | 4.939 | | 0.000147848 | | 0.000558203 | |  |
| 32 | hsa-miR-1280 | 1.325 | | 0.0002297 | | 0.000849889 | |  |
| 33 | hsa-miR-134 | 4.277 | | 0.000236397 | | 0.000868875 | |  |
| 34 | hsa-miR-31 | 1.914 | | 0.000265635 | | 0.000963579 | |  |
| 35 | hsa-miR-18a | 3.483 | | 0.000328063 | | 0.001159713 | |  |
| 36 | hsa-miR-106b | 1.634 | | 0.000354283 | | 0.00122892 | |  |
| 37 | ggo-miR-134 | 4.338 | | 0.000538641 | | 0.001834023 | |  |
| 38 | hsa-miR-574-3p | 3.251 | | 0.000753758 | | 0.002460799 | |  |
| 39 | ggo-miR-31 | 2.065 | | 0.000858484 | | 0.002770108 | |  |
| 40 | hsa-miR-3178 | 1.397 | | 0.00090595 | | 0.002888618 | |  |
| 41 | hsa-miR-197 | 1.633 | | 0.000910826 | | 0.002888618 | |  |
| 42 | hsa-miR-25 | 1.867 | | 0.001119293 | | 0.003509646 | |  |
| 43 | hsa-miR-1180 | 1.161 | | 0.001385913 | | 0.004135387 | |  |
| 44 | hsa-miR-181a | 1.535 | | 0.001576085 | | 0.004628187 | |  |
| 45 | hsa-miR-331-3p | 0.891 | | 0.002325579 | | 0.006551758 | |  |
| 46 | hsa-miR-543 | 6.879 | | 0.003237488 | | 0.00889508 | |  |
| 47 | ptr-miR-25 | 2.008 | | 0.003405394 | | 0.009310314 | |  |
| 48 | hsa-miR-4286 | 1.704 | | 0.003726849 | | 0.010089763 | |  |
| 49 | hsa-miR-4298 | 2.536 | | 0.003831073 | | 0.010321581 | |  |
| 50 | hsa-miR-182 | 2.136 | | 0.013409725 | | 0.029889146 | |  |
| 51 | hsa-miR-34c-3p | 5.266 | | 0.015069505 | | 0.03318879 | |  |
| 52 | hsa-miR-20a | 0.819 | | 0.018580192 | | 0.039509604 | |  |
| 53 | hsa-miR-1260b | 2.542 | | 0.020313244 | | 0.04237558 | |  |
| 54 | hsa-miR-378 | 1.817 | | 0.023665287 | | 0.047587806 | |  |

|  | **Table 5b: AGMK1-9T7 miRNAs whose expression was significantly decreased at p40 (q<0.05)** | | | |
| --- | --- | --- | --- | --- |
|  |  |  |  |  |
|  | **miRNA** | **Log2(p40v0 fold change)** | **p40v0 pval** | **p40v0 qval** |
| 1 | hsa-miR-451 | -12.655 | 6.11E-13 | 1.69E-10 |
| 2 | ggo-miR-10b | -9.109 | 1.91E-12 | 2.65E-10 |
| 3 | ggo-miR-141 | -9.364 | 6.73E-11 | 3.40E-09 |
| 4 | hsa-miR-10b | -5.419 | 1.06E-10 | 4.51E-09 |
| 5 | hsa-miR-1915 | -8.174 | 1.20E-10 | 4.76E-09 |
| 6 | hsa-miR-199a-5p | -6.630 | 1.54E-10 | 5.71E-09 |
| 7 | ppy-miR-638 | -3.652 | 1.04E-09 | 2.40E-08 |
| 8 | hsa-miR-145 | -6.404 | 1.38E-09 | 2.90E-08 |
| 9 | hsa-miR-30e* | -4.469 | 1.42E-09 | 2.90E-08 |
| 10 | hsa-let-7e | -2.957 | 3.42E-08 | 4.10E-07 |
| 11 | hsa-miR-660 | -3.688 | 6.25E-08 | 6.79E-07 |
| 12 | hsa-miR-1308 | -3.950 | 7.21E-08 | 7.55E-07 |
| 13 | hsa-miR-424 | -3.394 | 2.24E-07 | 1.95E-06 |
| 14 | hsa-miR-4328 | -8.971 | 3.89E-07 | 3.13E-06 |
| 15 | hsa-miR-30b | -1.308 | 5.43E-07 | 4.19E-06 |
| 16 | hsa-miR-30a | -1.730 | 9.30E-07 | 6.88E-06 |
| 17 | hsa-miR-513a-5p | -5.442 | 9.76E-07 | 7.03E-06 |
| 18 | ppy-miR-564 | -2.840 | 1.10E-06 | 7.80E-06 |
| 19 | hsa-miR-4281 | -3.078 | 6.94E-06 | 3.89E-05 |
| 20 | ggo-miR-30b | -1.316 | 8.31E-06 | 4.49E-05 |
| 21 | hsa-miR-557 | -4.646 | 1.05E-05 | 5.47E-05 |
| 22 | hsa-miR-19a | -2.866 | 1.41E-05 | 7.16E-05 |
| 23 | ggo-miR-214 | -4.986 | 2.08E-05 | 0.000103042 |
| 24 | hsa-miR-340 | -1.549 | 2.83E-05 | 0.000131842 |
| 25 | hsa-miR-30a* | -3.186 | 3.32E-05 | 0.000149979 |
| 26 | hsa-let-7b | -4.179 | 4.25E-05 | 0.000185746 |
| 27 | hsa-miR-30c | -1.265 | 5.00E-05 | 0.00021688 |
| 28 | hsa-miR-30d | -1.454 | 5.40E-05 | 0.000228924 |
| 29 | hsa-miR-2861 | -5.869 | 5.83E-05 | 0.000243411 |
| 30 | hsa-miR-671-5p | -3.844 | 7.05E-05 | 0.000285717 |
| 31 | hsa-miR-34a | -3.909 | 8.11E-05 | 0.00032383 |
| 32 | hsa-miR-1975 | -1.570 | 8.66E-05 | 0.000343128 |
| 33 | hsa-miR-193a-5p | -2.203 | 9.59E-05 | 0.000374831 |
| 34 | hsa-miR-200b | -8.680 | 0.000280189 | 0.001009774 |
| 35 | mml-miR-1227 | -2.790 | 0.000313328 | 0.001121916 |
| 36 | mml-miR-762 | -2.313 | 0.000315705 | 0.001123182 |
| 37 | hsa-miR-1275 | -2.072 | 0.000347004 | 0.001211241 |
| 38 | ppy-miR-1246 | -2.557 | 0.000675371 | 0.002271701 |
| 39 | hsa-miR-1228* | -5.032 | 0.000680047 | 0.002273653 |
| 40 | hsa-miR-4270 | -1.703 | 0.000908914 | 0.002888618 |
| 41 | hsa-miR-1293 | -7.365 | 0.001067937 | 0.003367643 |
| 42 | mml-miR-765 | -1.930 | 0.001233322 | 0.003813399 |
| 43 | mml-miR-638 | -1.689 | 0.001236778 | 0.003813399 |
| 44 | hsa-miR-663 | -5.942 | 0.00137908 | 0.004135387 |
| 45 | mml-miR-1230 | -3.251 | 0.001547669 | 0.004568915 |
| 46 | ppy-miR-298 | -5.239 | 0.001847008 | 0.005362789 |
| 47 | hsa-miR-200c | -7.256 | 0.001864898 | 0.005362789 |
| 48 | hsa-miR-4257 | -4.769 | 0.002307496 | 0.00653398 |
| 49 | hsa-miR-30e | -4.685 | 0.002995744 | 0.008313191 |
| 50 | hsa-miR-1246 | -2.194 | 0.003072297 | 0.008483207 |
| 51 | hsa-miR-126* | -8.880 | 0.003447983 | 0.009380541 |
| 52 | hsa-miR-214 | -5.588 | 0.004488454 | 0.011695268 |
| 53 | hsa-miR-194 | -4.860 | 0.005342354 | 0.013514292 |
| 54 | mml-miR-512-5p | -8.087 | 0.005357017 | 0.013514292 |
| 55 | ppy-miR-199b-3p | -6.983 | 0.005407492 | 0.013568745 |
| 56 | hsa-miR-100 | -2.808 | 0.006132554 | 0.015126966 |
| 57 | hsa-miR-215 | -6.636 | 0.006702978 | 0.016245208 |
| 58 | ggo-miR-224 | -4.456 | 0.007924237 | 0.018875329 |
| 59 | hsa-miR-192 | -3.980 | 0.007970376 | 0.018904098 |
| 60 | hsa-miR-940 | -2.524 | 0.009262915 | 0.021600495 |
| 61 | ppy-miR-1303 | -5.939 | 0.012589415 | 0.02851888 |
| 62 | hsa-miR-4289 | -3.035 | 0.014115617 | 0.03133667 |
| 63 | hsa-miR-152 | -6.741 | 0.014349655 | 0.031729316 |
| 64 | hsa-miR-711 | -5.736 | 0.01670429 | 0.035963876 |
| 65 | hsa-miR-373* | -4.383 | 0.016817546 | 0.036037599 |
| 66 | ppy-miR-1207-5p | -3.016 | 0.019132581 | 0.040374839 |
| 67 | ppy-miR-1268 | -4.261 | 0.021331553 | 0.044011197 |
| 68 | ggo-miR-145 | -6.975 | 0.025079269 | 0.049888868 |
